# Proteomes from AMPK-inhibited peripheral blood mononuclear cells suppress the progression of breast cancer and bone metastasis

**DOI:** 10.7150/thno.80294

**Published:** 2023-02-05

**Authors:** Kexin Li, Xun Sun, Kazumasa Minami, Keisuke Tamari, Kazuhiko Ogawa, Hudie Li, Hailan Ma, Meng Zhou, Sungsoo Na, Bai-Yan Li, Hiroki Yokota

**Affiliations:** 1 Department of Pharmacology, School of Pharmacy, Harbin Medical University, Harbin 150081, China; 2 Department of Biomedical Engineering, Indiana University Purdue University Indianapolis, Indianapolis, IN 46202, USA; 3 Department of Radiation Oncology, Osaka University Graduate School of Medicine; Suita, Osaka 565-0871, Japan; 4 Indiana Center for Musculoskeletal Health, Indiana University School of Medicine, Indianapolis, IN 46202, USA; 5 Simon Comprehensive Cancer Center, Indiana University School of Medicine, Indianapolis, IN 46202, USA

**Keywords:** breast cancer bone metastasis, PBMCs, conditioned medium, AMPK, Metadherin

## Abstract

**Background:** During a developmental process, embryos employ varying tactics to remove unwanted cells. Using a procedure analogous to some of the embryonic cells, we generated a tumor-eliminating conditioned medium (CM) from AMPK-inhibited lymphocytes and monocytes in peripheral blood mononuclear cells (PBMCs).

**Methods:** AMPK signaling was inhibited by the application of a pharmacological agent, Dorsomorphin, and the therapeutic effects of their conditioned medium (CM) were evaluated using *in vitro* cell cultures, *ex vivo* breast cancer tissues, and a mouse model of mammary tumors and tumor-induced osteolysis. The regulatory mechanism was evaluated using mass spectrometry-based proteomics, Western blotting, immunoprecipitation, gene overexpression, and RNA interference.

**Results:** While AMPK signaling acted mostly anti-tumorigenic, we paradoxically inhibited it to build induced tumor-suppressing cells and their tumor-eliminating CM. In a mouse model of breast cancer, the application of AMPK-inhibited lymphocyte-derived CM reduced mammary tumors additively to a chemotherapeutic agent, Taxol. It also prevented bone loss in the tumor-bearing tibia. Furthermore, the application of CM from the patient-derived peripheral blood diminished *ex vivo* breast cancer tissues isolated from the same patients. Notably, proteins enriched in CM included Moesin (MSN), Enolase 1 (ENO1), and polyA-binding protein 1 (PABPC1), which are considered tumorigenic in many types of cancer. The tumor-suppressing actions of MSN and ENO1 were at least in part mediated by Metadherin (Mtdh), which is known to promote metastatic seeding.

**Conclusion:** We demonstrated that PBMCs can be used to generate tumor-suppressive proteomes, and extracellular tumor-suppressing proteins such as MSN, ENO1, and PABPC1 are converted from tumor-promoting factors inside cancer cells. The results support the possibility of developing autologous blood-based therapy, in which tumor-suppressing proteins are enriched in engineered PBMC-derived CM by the inhibition of AMPK signaling.

## Introduction

Because of its aggressiveness, the overall survival of metastatic triple-negative breast cancer has not improved in the past few decades 1. While targeted drugs such as poly (ADP-ribose) polymerase inhibitors and immune checkpoint inhibitors have been developed, conventional chemotherapy, using highly cytotoxic agents such as Doxorubicin, Paclitaxel, and Cisplatin, is the mainstay 2. Because chemoresistance occurs in a sizable portion of patients, efforts have been directed at identifying novel therapeutic targets, including cell cycling, signaling kinases, angiogenesis, and epigenetic modifications 3. Here, we took an unconventional approach to generate induced tumor-suppressive cells (iTSCs) from peripheral blood samples and apply their conditioned medium (CM) for suppressing the progression of breast cancer.

The principle of generating iTSCs is counterintuitive. While both tumor cells and non-tumor cells can be converted into iTSCs, they are engineered by activating tumorigenic signaling or inhibiting anti-tumorigenic signaling 4-9. This unconventional approach was first validated with osteocytes 10. Although osteocytes could often present innate anti-tumor capabilities 11, the activation of Wnt signaling by the overexpression of Lrp5, a Wnt coreceptor, and β-catenin enhanced their anti-tumor action. Subsequently, the activation of Wnt signaling was found effective to convert other types of bone cells such as mesenchymal stem cells (MSCs), osteoblasts, and osteoclasts into iTSCs 5, 7, 8. Besides Wnt signaling, the activation of PI3K signaling was shown to generate MSC-driven iTSCs 12. The question was whether the activation of Wnt or PI3K signaling would be effective in other types of host cells into iTSCs. Here, a particular target was lymphocytes and monocytes, which can be isolated from peripheral blood mononuclear cells (PBMCs) of patients with breast cancer.

We have previously shown that the activation of cyclic AMP-activated protein kinase (PKA) signaling, and not Wnt/PI3K, can convert T lymphocytes into iTSCs 13, 14. In this study, instead of activating tumorigenic signaling such as PKA, we examined the possibility of 5'-AMP-activated protein kinase (AMPK) signaling in the generation of iTSCs. AMPK is involved in a variety of cellular activities, but its effect on cancer progression is controversial 15. Its activation is reported to induce resistance to cytotoxicity 16, whereas it is also shown to negatively regulate mTOR signaling and suppress the proliferation of cancer cells 17. While AMPK signaling can be a double-edged sword, we examined whether its activation or inactivation could generate iTSCs and tumor-suppressive CM.

In this study, we conducted *ex vivo* assays using cancer tissues and peripheral blood samples, both of which were isolated from the same patients with breast cancer. While peripheral blood contains varying types of cells, we focused on PBMCs that were mainly composed of lymphocytes and monocytes. Besides triple-negative breast cancer, we evaluated the efficacy of the autologous blood-derived CM with 3 breast cancer subtypes, including ER^+^PR^+^HER2^-^, ER^+^PR^-^HER2^-^, and ER^-^PR^+^HER2^+^. The blood samples from healthy volunteers were also employed to generate tumor-suppressive CM. In evaluating a novel therapeutic option, one of the topics is its compatibility with existing treatments. Focusing on Taxol, a popularly subscribed chemo drug for triple-negative breast cancer and metastatic breast cancer 18, we evaluated its IC_50_ with and without a co-administration of the engineered CM and tested whether CM could additively assist in the tumor-inhibitory action of Taxol.

The unique feature of the described iTSC approach is the double-edged role of tumor-suppressing proteins. Extracellular tumor-suppressing proteins, which are enriched in the engineered CM, are generally tumorigenic in breast cancer cells. For example, global proteomics analysis previously revealed that Enolase 1 (ENO1) and Moesin (MSN) acted as extracellular tumor-suppressing proteins in MSC-derived CM 5. Notably, both of them were considered tumorigenic in many types of cancer 19, 20. In the present study, ENO1 and MSN were enriched also in lymphocyte-derived CM. They exerted their anti-tumor actions at least in part via Metadherin (Mtdh), which is recognized as a therapeutic target of multiple cancers including breast cancer 21. Remarkably, the overexpression of Mtdh in lymphocytes converted them into iTSCs. Besides ENO1 and MSN, the engineered CM was enriched with polyadenylate-binding protein 1 (PABPC1) which also showed an opposing tumor-regulating role inside and outside tumor cells. CAR-T immunotherapy engineers lymphocytes and strengthen their anti-tumor capabilities by regulating the interactions of PDL1 with PD1 on T cells. Here, we examined the effect of the described lymphocyte-derived CM on the expression of PDL1 22. Collectively, we demonstrated the iTSC-based therapeutic option with PBMCs and highlighted distinctively different aspects of the proteomes across the plasma membrane.

## Results

### Generation of tumor-suppressive CM using lymphocytes

**Figure 1:**
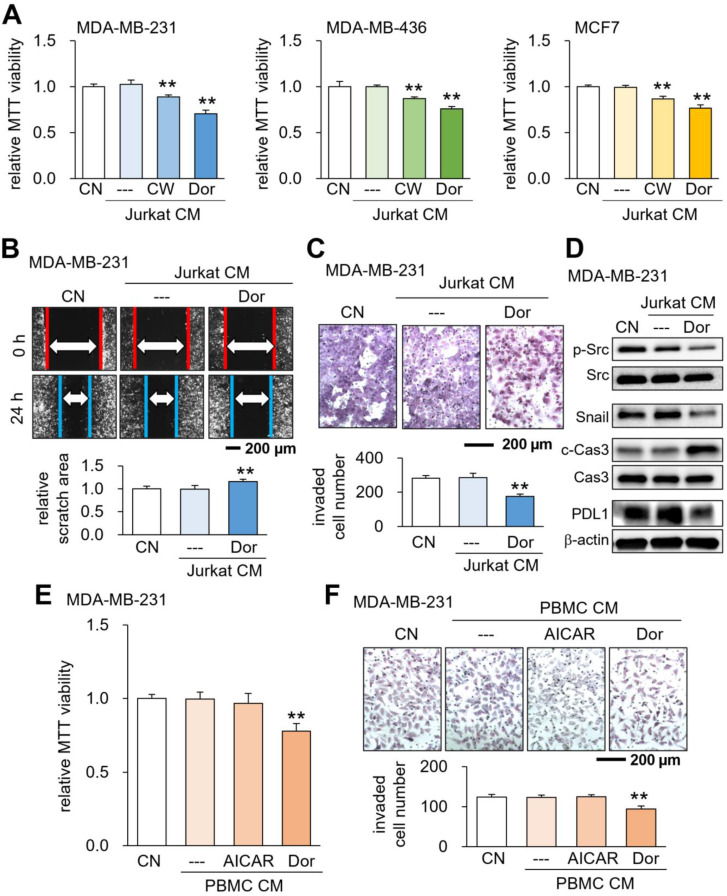
** Suppression of tumorigenic behaviors of breast cancer cells by CM^Lym-Dor^.** CN = control, CW = CW008 (PKA activator), Dor = Dorsomorphin (AMPK inhibitor), AICAR (AMPK activator), PBMC = peripheral blood mononuclear cells, and CM = conditioned medium. The double asterisks indicate p < 0.01. (A) Suppression of MTT-based viability of MDA-MB-231, MDA-MB-436, and MCF7 breast cancer cells by CM^Lym-CW^ and CM^Lym-Dor^, respectively. (B&C) Suppression of scratch-based migration, and transwell invasion of MDA-MB-231 cells by CM^Lym-Dor^. (D) Reduction in p-Src, Snail, and PDL1, together with the elevation in c-Cas3 (cleaved caspase 3) in MDA-MB-231 cells by CM^Lym-Dor^. (E&F) Suppression of MTT-based viability, and transwell invasion of MDA-MB-231 cells by CM^PBMC-Dor^, which was derived from peripheral blood mononuclear cells with Dorsomorphin.

Using Jurkat T lymphocytes, we generated tumor-suppressive CM by activating PKA signaling as well as inhibiting AMPK signaling. The activation of PKA signaling by 20 μM CW008, a pharmacological activator of PKA, generated CM^Lym-CW^ that reduced MTT-based viability of MDA-MB-231 breast cancer cells, as well as MDA-MB-436 and MCF-7 breast cancer cell lines (Fig. 1A). Analogously, the inactivation of AMPK signaling by 50 μM Dorsomorphin (Dor) generated CM^Lym-Dor^ that decreased the cellular viability of these cancer cell lines (Fig. 1A). Also, CM^Lym-Dor^ suppressed 2-dimensional motility, transwell invasion capacity, and EdU-based proliferation of MDA-MB-231 cells (Fig. 1B&C, Supplementary Fig. 1A). The viability of EO771 and 4T1.2 mammary tumor cells was also reduced by CM^Lym-Dor^ and CM^Lym-CW^ (Supplementary Fig. 1B). We also observed the suppression of MTT-based viability by Dorsomorphin-treated J774A.1 monocyte-derived CM^mono-Dor^ (Supplementary Fig. 1C). In MDA-MB-231 cells, the tumor-suppressive actions of CM^Lym-Dor^ were associated with the downregulation of p-Src, Snail, and PDL1, together with the upregulation of caspase 3, an apoptotic marker (Fig. 1D). The MTT-based viability and transwell invasion were also suppressed by CM, derived from Dorsomorphin-treated PBMCs (Fig. 1E&F). By contrast, the treatment of lymphocytes and PBMCs with AICAR, an activator of AMPK, did not generate tumor-suppressive CM (Fig. 1E&F, Supplementary Fig. 2). In the *ex vivo* assay using freshly isolated human breast cancer tissues of three subtypes such as ER+PR+HER2-, ER+PR-HER2-, and ER-PR+, HER2+, CM^Lym-Dor^ significantly diminished the fragment size in 4 days (Supplementary Fig. 3).

In this study, we focused on the proteomics of CM^Lym-Dor^ and did not evaluate the potential effects of DNA and RNA in the secretome, since the incubation with nuclease-treated CM^Lym-Dor^ did not significantly alter their anti-tumor effect on MDA-MB-231 cells (Supplementary Fig. 4).

### Opposing effects of AMPK signaling on tumor cells indirectly via lymphocytes and directly to tumor cells

**Figure 2:**
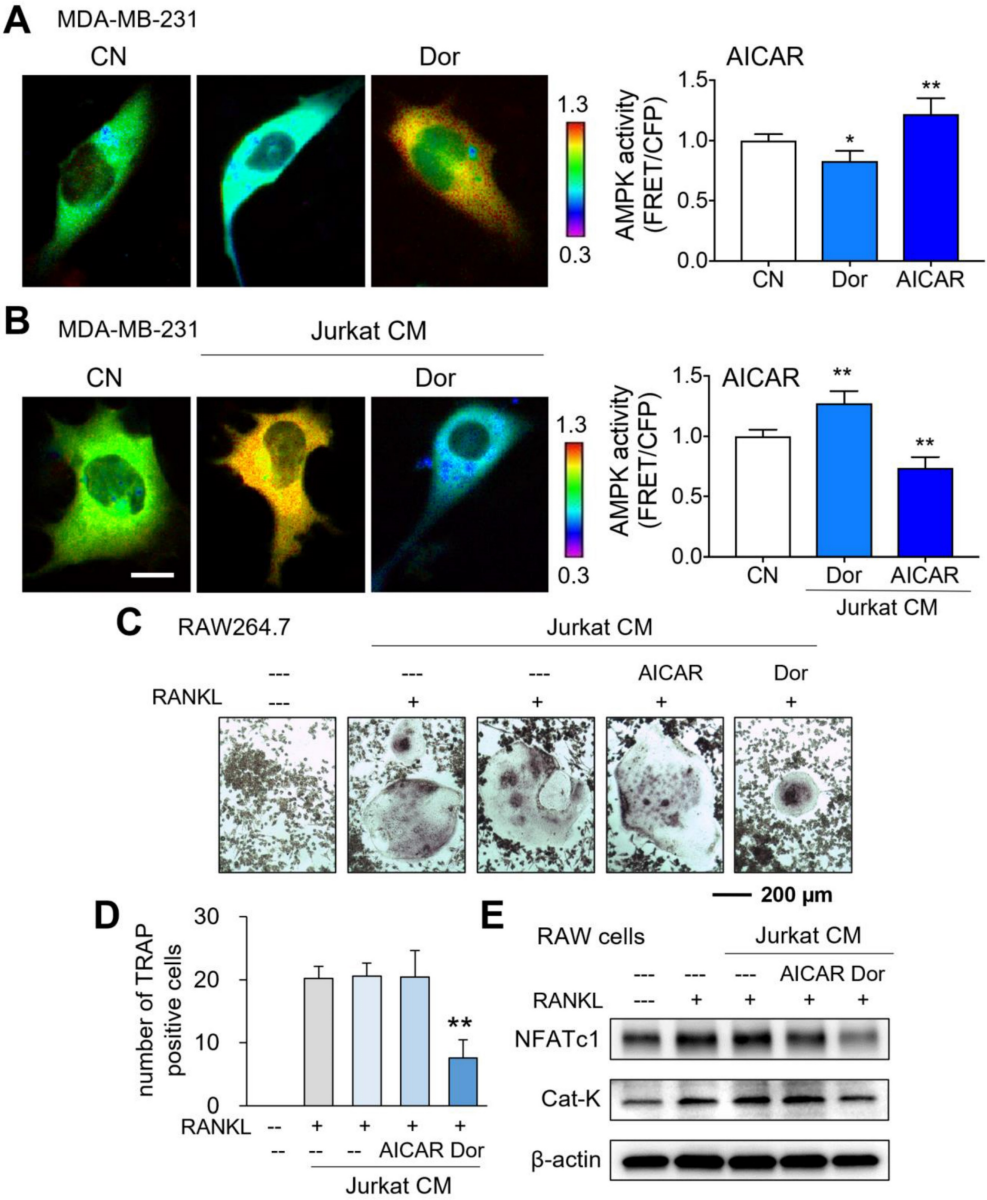
** Opposing effects of AMPK signaling directly and via lymphocytes on MDA-MB-231 breast cancer cells, and the inhibitory role of CM^Lym-Dor^ in osteoclastogenesis.** CN = control, Dor = Dorsomorphin (AMPK inhibitor), and CM = conditioned medium. The single and double asterisks indicate p < 0.05 and 0.01, respectively. (A) Live-cell FRET images for reduction and elevation in AMPK activity by the treatment with Dorsomorphin and AICAR, respectively. (B) Live-cell FRET images for elevation and reduction in AMPK activity by CM^Lym-Dor^ and CM^Lym-AICAR^, respectively. Of note, Dorsomorphin and AICAR are the inhibitor and activator of AMPK signaling, respectively. (C&D) Suppression of the TRAP (tartrate-resistant acid phosphatase)-positive multi-nucleation of RANKL-treated RAW264.7 pre-osteoclasts by CM^Lym-Dor^. (E) Reduction in NFATc1 and Cat K by CMLym-Dor and no detectable effects of CM^Lym-AICAR^ in RAW264.7 cells.

While AMPK-activated cell-derived CM presented tumor-suppressive capabilities, we next evaluated AMPK activities using a fluorescence resonance energy transfer (FRET)-based biosensor. As expected in live-cell imaging, Dorsomorphin decreased the AMPK activity of MDA-MB-231 breast cancer cells, while AICAR increased it (Fig. 2A). However, the responses of MDA-MB-231 cells became opposite when they were exposed to Dor or AICAR-treated lymphocyte-derived CM. CM^Lym-Dor^ elevated the activity of AMPK in MDA-MB-231 cells, whereas CM^Lym-AICAR^ reduced it (Fig. 2B). Collectively, the opposing effects on AMPK signaling were observed in MDA-MB-231 cells in response to the activator and inhibitor of AMPK, depending on whether Dorsomorphin and AICAR were applied directly to cells or indirectly through iTSC CM.

### Inhibition of osteoclast development by Dorsomorphin-treated CM (CM^Lym-Dor^)

Besides the anti-tumor action, we examined the effect of CM^Lym-Dor^ on the development of bone-resorbing osteoclasts. RAW264.7 pre-osteoclasts were stimulated for differentiation by 40 ng/mL of RANKL and their multi-nucleation was induced. In response to CM^Lym-Dor^, the multinucleation was significantly reduced together with the decrease in the levels of NFATc1, a transcription factor for osteoclastogenesis, and Cathepsin K (Cat K), a potent proteinase for cleaving bone matrix proteins (Fig. 2C-E). By contrast, AICAR-treated CM did not block osteoclast development without detectably changing the RANKL-driven increase in NFATc1 and Cathepsin K.

### Reduction in mammary tumors and osteolysis in the tibia

**Figure 3:**
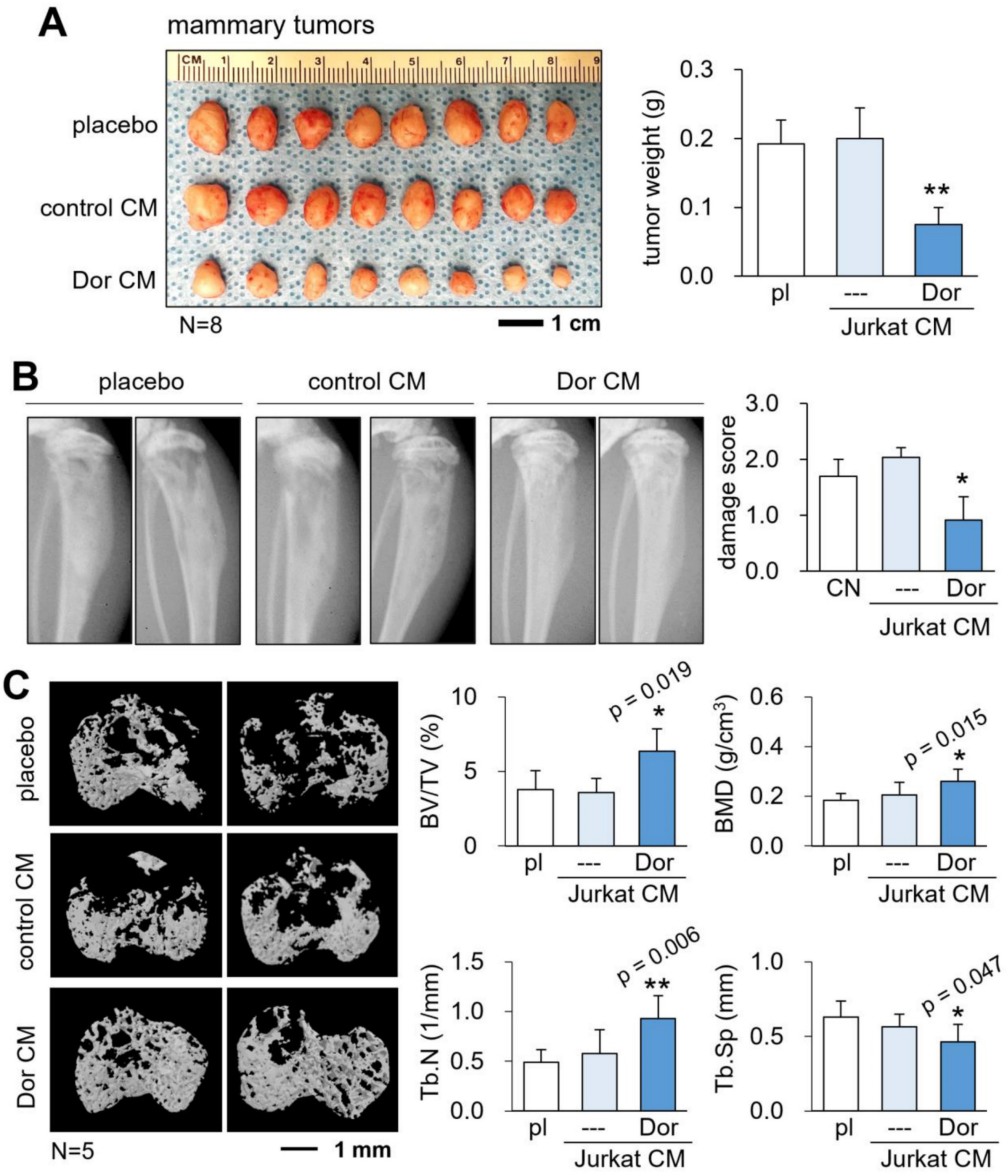
** Tumor suppression by CM^Lym-Dor^ in a mouse model of the primary tumor and bone metastasis.** pl = placebo, CM = conditioned medium, and Dor = Dorsomorphin (AMPK inhibitor). The single and double asterisks indicate p < 0.05 and 0.01, respectively. (A) Reduction in the size and weight of mammary tumors by CM^Lym-Dor^ in NSG (NOD/SCID/γ [-/-]) female mice. (B) Reduction in damages in X-ray images by CM^Lym-Dor^. (C) Elevation of bone volume ratio (BV/TV), bone mineral density (BMD), and trabecular number (Tb.N), as well as the decrease in trabecular serration (Tb.Sp) by CM^Lym-Dor^.

So far, we showed the *in vitro* tumor-suppressing, anti-osteoclastogenic actions of CM^Lym-Dor^. Next, we evaluated the *in vivo* role of CM^Lym-Dor^ using an NSG mouse model of mammary tumors and tumor invasion to the proximal tibia. The daily administration of CM^Lym-Dor^ as an intravenous injection from the tail vein for 18 days significantly reduced the size and weight of mammary tumors (N = 8; Fig. 3A). Also, the systemic injection of CM^Lym-Dor^ daily for 18 days suppressed the tumor-driven damage of the tibia in X-ray images (N = 5, Fig. 3B), in which the damage score of 0, 1, 2, and 3 indicated normal, slight, medium, and severe damages, respectively. The microCT images revealed that the administration of CM^Lym-Dor^ prevented bone loss in the tumor-invaded proximal tibia (N = 5, Fig. 3C). Consistently, the bone volume ratio (BV/TV), bone mineral density (BMD), and trabecular number (Tb.N) were elevated by the administration of CM^Lym-Dor^, with a decrease in trabecular separation (Tb.Sp).

### Additive antitumor effect with Taxol

**Figure 4:**
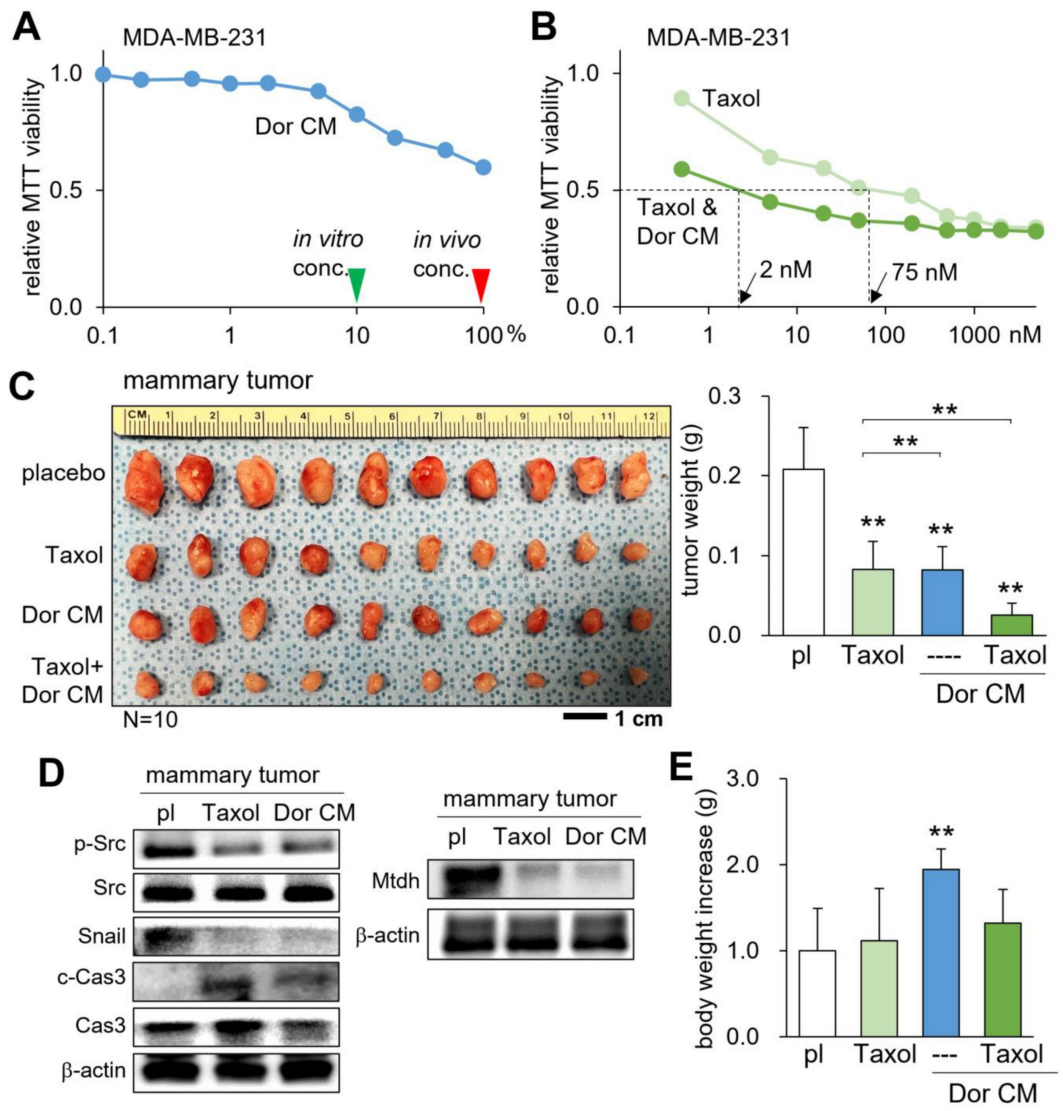
**Additive tumor-suppressing effects by Taxol and CM^Lym-Dor^ in a mouse model of the primary tumor.** pl = placebo, Dor = Dorsomorphin (AMPK inhibitor), and CM = conditioned medium. The double asterisk indicates p < 0.01. (A) Dose responses of CM^Lym-Dor^. The green and red arrowheads indicate the concentration of CM^Lyn-Dor^ for the *in vitro* and *in vivo* experiments, respectively. (B) Dose responses of Taxol with and without CM^Lym-Dor^. The doses of 2 nM and 75 nM are IC50 (50% inhibition) for Taxol with and without CM^Lyn-Dor^. (C) Additive reduction in the size and weight of mammary tumors by Taxol and CM^Lym-Dor^ in NOD/SCID/γ (-/-) (NSG) female mice. (D) Reduction in p-Src and Snail, Mtdh, and the elevation in c-Cas3 (cleaved caspase 3) in the tumor tissues in tumor-inoculated mice that were treated with Taxol or CM^Lym-Dor^. (E) Increase in body weight by CM^Lym-Dor^.

Taxol (Paclitaxel) is a chemotherapeutic drug that has been frequently administered for the treatment of breast cancer in adjuvant and neoadjuvant therapy, as well as metastatic breast cancer. We examined whether CM^Lym-Dor^ can suppress the progression of mammary tumors additively with Taxol. The result of the MTT-based cell viability assay revealed that Taxol's IC_50_ of 75 nM was lowered to 2 nM when given simultaneously with CM^Lym-Dor^ (Fig. 4A&B). In the mouse model, the weekly injection of Taxol, as well as the daily injection of CM^Lym-Dor^ reduced the size and weight of mammary tumors (Fig. 4C). Notably, the simultaneous administration of Taxol and CM^Lym-Dor^ further reduced the tumor progression. In the mammary tumor, the levels of p-Src, Snail, and Mtdh were decreased, while the level of caspase 3 was increased (Fig. 4D). We also observed that the CM-administered group presented a significant increase in body weight, indicating that CM^Lym-Dor^ did not induce appetite loss (Fig. 4E) that is in many cases associated with the treatment with chemotherapeutic agents.

### Generation of CM from patient-derived peripheral blood cells

**Figure 5:**
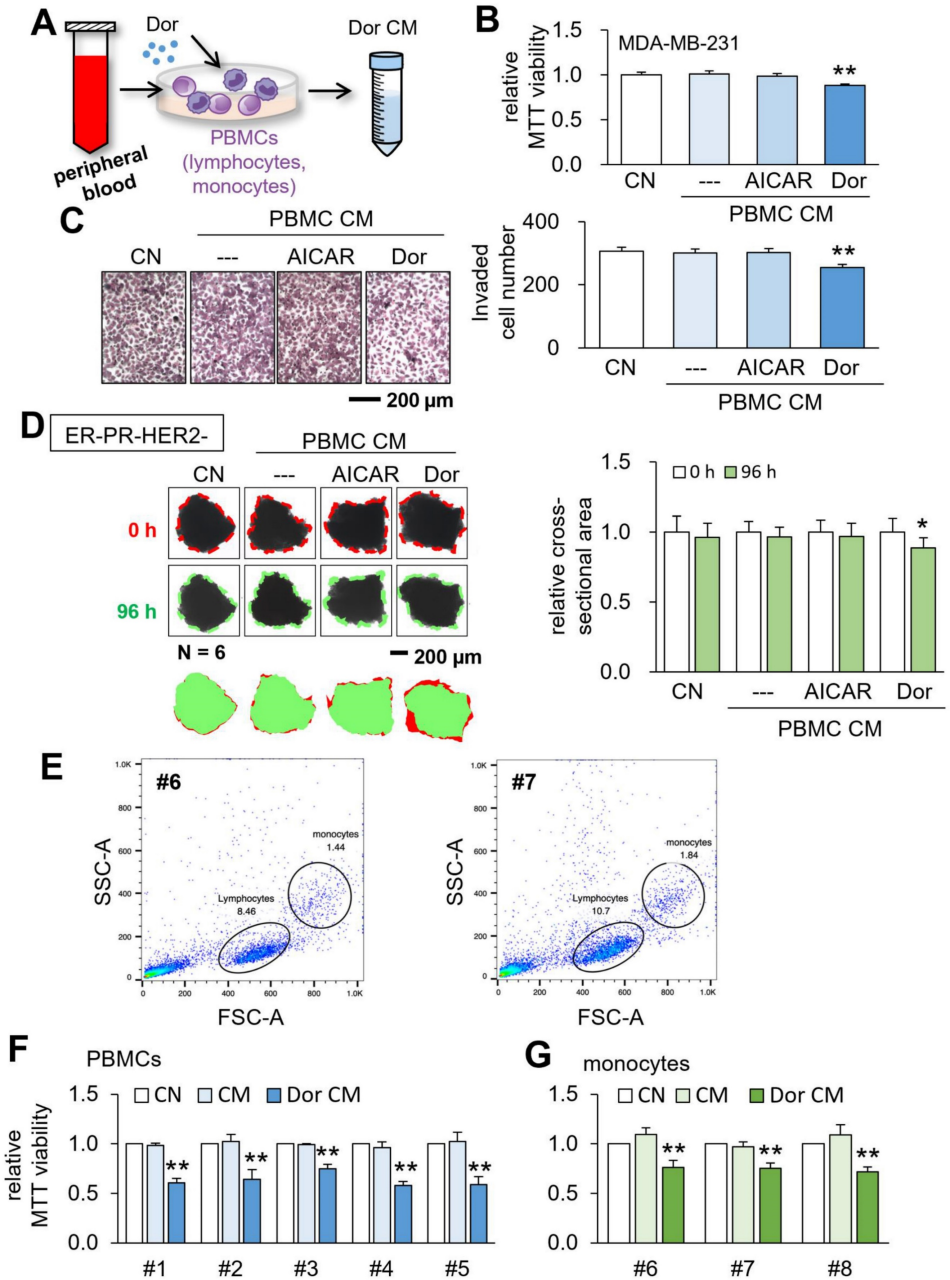
** Tumor suppression by CM, which was derived from the peripheral blood.** CN = control, CM = conditioned medium, PBMCs = peripheral blood mononuclear cells, and Dor = Dorsomorphin (AMPK inhibitor). The single and double asterisks indicate p < 0.05 and 0.01, respectively. (A) Procedure to generate tumor-suppressive CM from the peripheral blood. (B&C) Reduction in MTT-based cell viability and transwell invasion of MDA-MB-231 breast cancer cells by CM, which was derived from a patient with triple-negative breast cancer (TNBC). The bar (---) in the second column designated DMSO-treated PBMC-derived CM, which served as a negative control. (D) Shrinkage of human breast cancer tissue fragments (ER-PR-/HER2-) by CM^PBMC-Dor^. (E) Flow cytometry analysis of the peripheral blood samples (HV46 and HV47) using forward scatter (FSC) and side scatter (SSC). (F) Reduction in MTT-based cell viability of MDA-MB-231 breast cancer cells by CM^PBMC-Dor^, which was derived from five independent samples. (G) Reduction in MTT-based cell viability of MDA-MB-231 breast cancer cells by CM^mono-Dor^, which was derived from three independent samples.

Based on the tumor-suppressive capability of Jurkat lymphocyte-derived CM, we next generated CM from the blood samples of patients with breast cancer (Fig. 5A). Human PBMCs were isolated from a patient with triple-negative breast cancer, and CM^PBMC-Dor^ and CM^PBMC-AICAR^ were generated. Of note, CM^PBMC-AICAR^ served as a negative control. Using MDA-MB-231 breast cancer cells, we observed that CM^PBMC-Dor^ suppressed MTT-based viability and transwell invasion, while CM^PBMC-AICAR^ did not present any detectable changes (Fig. 5B&C). We also conducted the *ex vivo* assay using freshly isolated triple-negative breast cancer tissues, in which CM was generated from PBMCs of the tissue provider. The incubation of the manually dissected fragments (~300 μm long) with CM^PBMC-Dor^ for 4 days significantly decreased the mean fragment size (Fig. 5D). Besides triple-negative breast cancer, CM^PBMC-Dor^ was also generated using the blood sample from a patient with ER^+^PR^+^HER2^-^, and we observed that it reduced fragment sizes of the cancer tissue from the same patient (Supplementary Fig. 5).

### Generation of CM from PBMCs and monocytes

Besides employing the blood samples from patients with breast cancer, we generated tumor-suppressive CM using the peripheral blood samples from healthy volunteers. Using the forward-scattered light (FSC) which is proportional to the cell-surface area and side-scattered light (SSC) which is linked to cell granularity, lymphocytes and monocytes were identified, and specifically, monocytes were isolated (Fig. 5E). Using PBMCs and monocytes, we generated CM^PBMC-Dor^ and monocyte-derived CM^mono-Dor^ from 5 and 3 independent blood samples, respectively. The MTT assay revealed that all CMs significantly reduced the viability of MDA-MB-231 breast cancer cells (Fig. 5F&G).

### Global proteomics analysis of CM^Lym-Dor^

**Figure 6:**
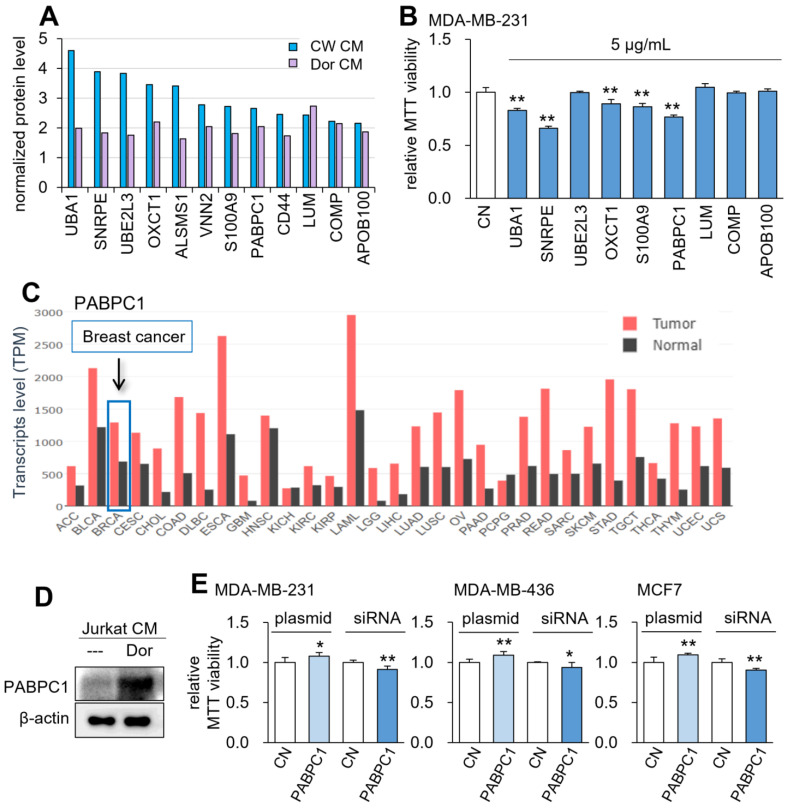
**Prediction of the tumor suppressors in CM by mass spectrometry-based whole-genome proteomics.** The single and double asterisks indicate p < 0.05 and 0.01, respectively. CN = control, CM = conditioned medium, CW = CW008 and Dor = Dorsomorphin. (A) The normalized protein level of the potential tumor suppressors by mass spectrometry-based whole-genome proteomics. (B) Reduction in MTT-based viability of MDA-MB-231 cells in response to 9 tumor-suppressing protein candidates (5 µg/ml) in 48 h. (C) PABPC1 expression profiles for all types of cancer and control tissues. (D) Western blot images, showing that the level of PABPC1 was elevated in CM^Lym-Dor^. (E) Elevation in MTT-based viability of MDA-MB-231, MDA-MB-436, and MCF-7 breast cancer cell lines by the overexpression of PABPC1, as well as its reduction by the silencing of PABPC1.

To determine the effective tumor-suppressing proteins for the anti-tumor action of CM^Lym-Dor^ and CM^Lym-CW^, mass spectrometry-based proteomics analysis was conducted. Among 50 proteins that were enriched each in CM^Lym-Dor^ and CM^Lym-CW^ (Table S1), we selected 9 protein candidates that were commonly enriched in the two CMs (Fig. 6A). Among them, 5 proteins, given at 5 µg/mL, induced a statistically significant decrease in viability of MDA-MB-231 cells in 2 days (Fig. 6B). Particularly, PABPC1 presented the second highest reduction in the MTT assay next to small nuclear ribonucleoprotein polypeptide E (SNRPE), and its transcript level was elevated in many cancer types including breast cancer (Fig. 6C). Consistent with the mass spectrometry data, the protein level of PABPC1 was elevated in CM^Lym-Dor^ (Fig. 6D). Furthermore, in MDA-MB-231 as well as MDA-MB-436 and MCF-7 breast cancer cells, the MTT-based viability was increased by the overexpression of PABPC1 and reduced by the silencing of PABPC1 (Fig. 6E, Supplementary Fig. 6A).

### Role of the ENO1/MSN-Mtdh regulatory axis

**Figure 7:**
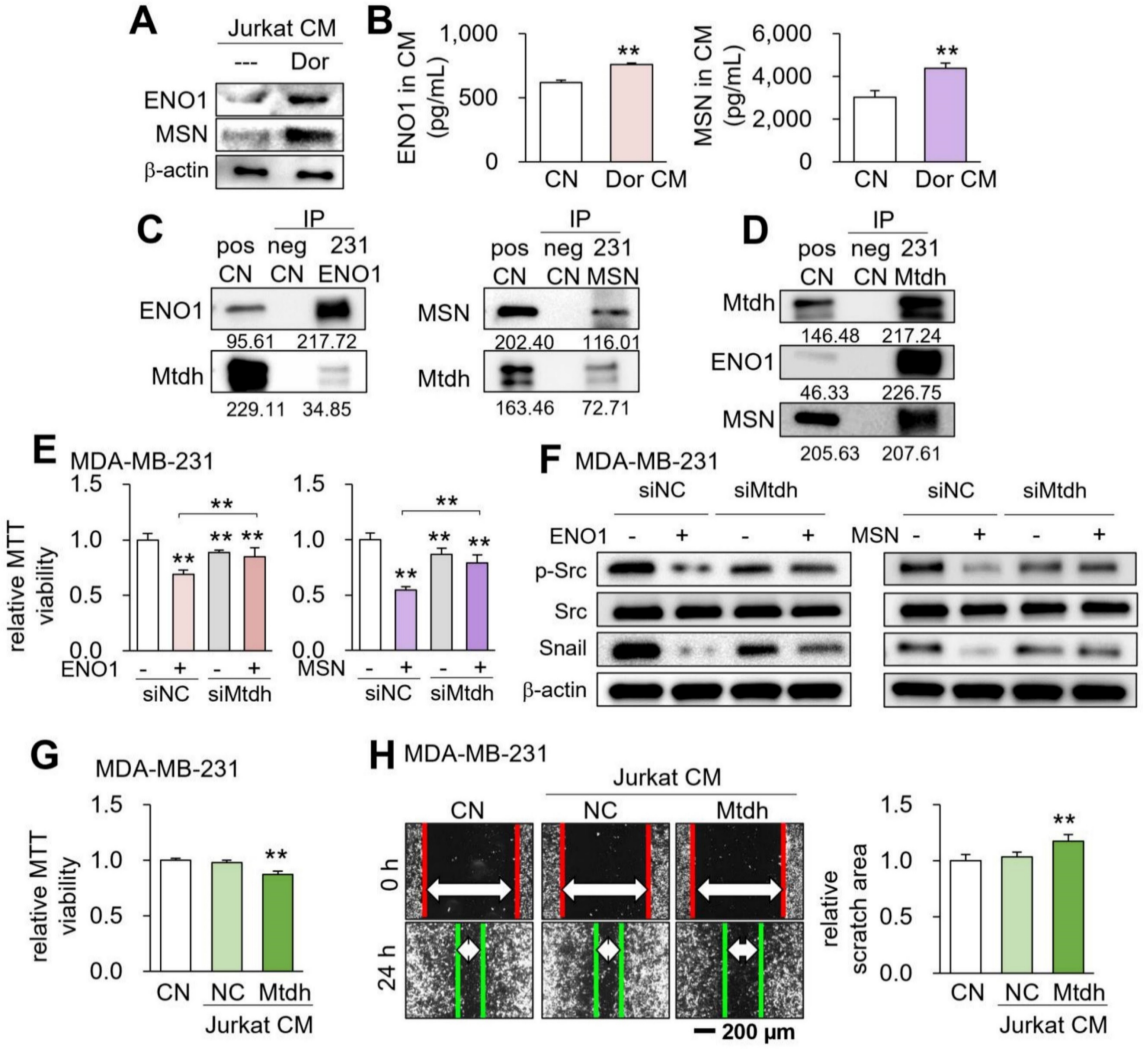
** Role of the Enolase 1 (ENO1)/Moesin (MSN)-Metadherin (Mtdh) regulatory axis.** CN = control, CM = conditioned medium, NC = negative control, si = siRNA, and Dor = Dorsomorphin. The double asterisk indicates p < 0.01. (A&B) Elevated levels of ENO1 and MSN in CM^Lym-Dor^. (C&D) Immunoprecipitation of Mtdh by ENO1 and MSN. (E) Reduction in the efficacy of ENO1 and MSN in Mtdh-silenced MDA-MB-231 cells. (F) Reduction of p-Src and Snail by the application of ENO1 and MSN recombinant proteins, and its suppression by silencing Mtdh. (G&H) Reduction in MTT-based cell viability and scratch-based migration of MDA-MB-231 breast cancer cells by Mtdh overexpressing Jurkat cell-derived CM.

We have previously shown that ENO1 and MSN are enriched in MSC-derived tumor-suppressive CM 5. In the present study, the levels of ENO1 and MSN were also elevated in CM^Lym-Dor^ (Fig. 7A&B). Mass spectrometry-based proteomics using MDA-MB-231 protein lysates predicted that 34 and 44 cell-membrane proteins could potentially interact with ENO1 and MSN, respectively (Table S2). Among them, two proteins, such as voltage-dependent anion-selective channel 1 (Vdac1) and Mtdh, gave a significantly low survival rate for patients with breast cancer in the TCGA database (Table S2). We selected Mtdh for our further analysis hereafter since its association with triple-negative breast cancer was reported 23, 24.

The reciprocal immunoprecipitation assay using MDA-MB-231 cell lysates supported the possible interaction of Mtdh with ENO1 and MSN (Fig. 7C&D). Furthermore, the reduction in MTT-based viability of MDA-MB-231 cells by ENO1 and MSN recombinant proteins was suppressed by silencing Mtdh (Fig. 7E, Supplementary Fig. 6B), while ENO1/MSN-driven downregulation of p-Src and Snail was also repressed by Mtdh siRNA (Fig. 7F). Notably, we observed that CM derived from Mtdh-overexpressing Jurkat T lymphocytes also presented the anti-tumor capabilities (Fig. 7G&H). This result was consistent with the notion that the activation of tumorigenic pathways can generate tumor-suppressing CM. In addition, we have previously shown that extracellular ENO1 and MSN interact with CD44 antigen 4, 6. Consistently, the decrease in MDA-MB-231 cell viability by ENO1 and MSN was reduced by silencing CD44, while the reduction of p-Src and Snail by ENO1 and MSN was suppressed by CD44 siRNA (Supplementary Fig. 7). Of note, ENO1 and MSN may bind to other proteins besides Mtdh. Compared to the intensity of ENO1, for instance, the low intensity of Mtdh using the cell lysate of MDB-MB-231 cells (Fig. 7C) indicates that ENO1 may have other binding partners and their binding to ENO1 is competitive to the binding of Mtdh to ENO1.

**Figure 8:**
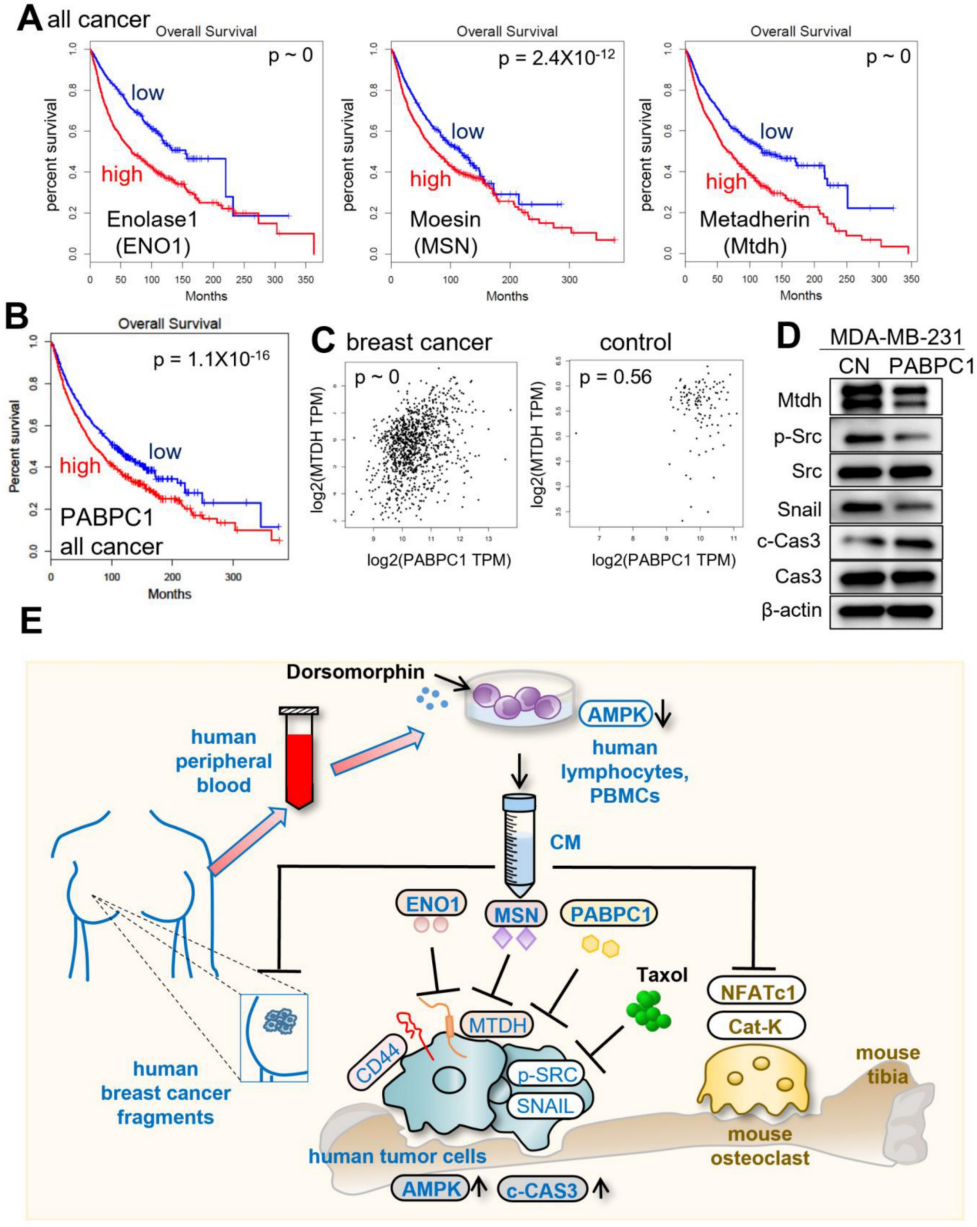
** Putative regulatory mechanism for the action of CM^Lym-Dor^**. (A) Reduced %survival of all types of cancer patients with high mRNA levels of ENO1, MSN, and Mtdh. (B) Lowered survival rates of all types of cancer patients with high levels of PABPC1. (C) Pairwise gene expression correlation analysis of PABPC1/MTDH in breast cancer and control. (D) Reduction of Mtdh, p-Src, and Snail with an elevation of cleaved caspase 3 (c-Cas3) in MDA-MB-231 cells by 5 µg/mL PABPC1 recombinant protein. (E) The schematic diagram for the regulatory mechanism of tumor-suppressing action of CM^Lym-Dor^. Human and mouse samples/molecules are shown in blue and brown, respectively.

Lastly, we examined the impact of the expression of ENO1, MSN, Mtdh, and PABPC1 on the survival rate of cancer patients. In the TCGA database, a high transcript level of Mtdh was linked to a poor survival rate (p ~ 0). However, the high transcript levels of ENO1, MSN, and PABPC1 also led to a poor survival rate (p ~ 0, 2.4x10^-12^, and 1.1x10^-16^, respectively) since they acted as tumor-suppressing proteins in CM (Fig. 8A&B). The results indicated that ENO1, MSN, and PABPC1 were double-edged swords, acting as intracellular tumor promoters and extracellular tumor suppressors. In the TCGA database, the transcript level of PABPC1 was positively correlated with that of Mtdh in breast cancer tissues (p ~ 0), while this correlation was weakened in control tissues (p = 0.56) (Fig. 8C). The application of 5 µg /mL of extracellular PABPC1 to MDA-MB-231 cells reduced the levels of Mtdh, p-Src, and Snail with the elevated level of cleaved caspase 3 (Fig. 8D), indicating the linkage of the ENO1/MSN-Mtdh regulatory axis with PABPC1.

## Discussion

This study presents the tumor-suppressive action of AMPK-inhibited PBMC/lymphocyte-derived CM and evaluates the role of ENO1/MSN-Mtdh regulatory axis as well as PABPC1 in suppressing the progression of breast cancer (Fig. 8E). FRET-based live-cell imaging showed that the application of Dorsomorphin, a pharmacological inhibitor of AMPK signaling, to breast cancer cells reduced the FRET-based AMPK activity, whereas Dorsomorphin-treated lymphocyte-derived CM promoted AMPK activity in breast cancer cells. In the mouse model, the engineered CM was effective in inhibiting the growth of mammary tumors as well as tumor-driven osteolysis in the tibia. The efficacy of CM was additive to Taxol, and *in vitro* IC_50_ was reduced by the simultaneous application of CM from 75 nM to 2 nM. Importantly, tumor-suppressive CM could be generated using the peripheral blood samples of patients with breast cancer as well as healthy individuals. Paradoxically, CM was enriched with PABPC1, ENO1, and MSN, all of which have been considered oncogenic since their elevated transcript levels in cancer tissues significantly reduce the survival rate. Besides CD44, the tumor-suppressive action of extracellular ENO1 and MSN was mediated by Mtdh. Consistently, we previously showed that the control CM gained tumor-suppressive capability by the addition of ENO1 6. The extracellular application of ENO1 recombinant proteins reduced the EdU-based proliferation and transwell invasion of mammary tumor cells, and it downregulated Lrp5, MMP9, Runx2, TGFβ, and Snail 6.

The generation of iTSCs can be carried out using multiple types of tumor and non-tumor cells including osteoblasts, osteocytes, MSCs, macrophages, and peripheral blood cells such as PBMCs, lymphocytes, and monocytes 25. However, little is known about the most effective procedure for each type of iTSC-generating cells. The activation of PI3K and Wnt pathways can generate iTSCs from MSCs but not from PBMCs and lymphocytes. We previously showed that the activation of PKA signaling converted lymphocytes into iTSCs 14. In this study, we revealed that besides PKA activation, AMPK inhibition by Dorsomorphin can generate iTSCs from PBMCs, lymphocytes, and monocytes. By contrast, the activation of AMPK by AICAR and the generation of AICAR-treated PBMC-derived CM did not significantly alter the viability or transwell invasion of breast cancer cells. We also evaluated the direct effect of Dorsomorphin and CW008 on tumor cells, together with other treatments that are reported to generate tumor-suppressive CM. Those additional treatments included the application of YS49 as a PI3K activator, as well as the overexpression of tumorigenic genes, such as Lrp5, β-catenin, cMyc, and Akt. The direct effects on tumor cells differed depending on the treatment and tumor cell lines, including MDA-MB-231, and MCF7, as well as U2OS osteosarcoma, MG63 osteosarcoma, and PANC1 pancreatic cancer. Particularly, the treatment with CW008 tended to reduce cell viability in breast cancer and pancreatic cancer cells except for osteosarcoma cells, while the treatment with Dorphomorphin showed a reduction in cell viability in the four cell lines except for pancreatic cancer cells (Supplementary Fig. 8). Importantly for osteolytic bone metastasis associated with breast cancer, AMPK-inactivated lymphocyte-derived CM suppressed the maturation of bone-resorbing RAW pre-osteoclasts. The activation of AMPK by AICAR did not inhibit the differentiation of RAW cells. Taken together, existing evidence supported the general rule that iTSCs can be engineered by activating tumorigenic signaling or inactivating anti-tumorigenic signaling.

Among the nine selected tumor-suppressing protein candidates that were enriched in lymphocyte-derived iTSC CM, five of them, including Uba1 (ubiquitin-like modifier activating enzyme 1), SNRPE (small nuclear ribonucleoprotein polypeptide E), OXCT1 (3-oxoacid CoA-transferase 1), S100A9 (S100 calcium-binding protein A9), and PABPC1 (polyadenylate-binding protein 1), reduced MTT-based viability of MDA-MB-231 breast cancer cells. We focused on PABPC1 that gave the most significant negative effect on the survival rate of all cancer patients (p = 1.1x10^-16^) and the second-strongest anti-tumor capability next to SNRPE. PABPC1 is known as a prognostic biomarker for gastric cancer and ovarian cancer 26, 27. It is mostly located in the cytoplasm and interacts with translation initiation factors such as EIF4A1, 4B, C1, etc. 28, but it is also reported to stimulate cell migration at the leading edge of cell projection and lamellipodium 29. In triple-negative breast cancer, SNRPE is reported highly expressed and it promotes tumor progression 30. SNRPE are mostly located in the cytoplasm and nucleus, and the mechanism of their anti-tumor actions requires further analysis.

Besides PABPC1, lymphocyte-derived iTSC CM had elevated levels of MSN and ENO1, which were enriched in MSC-derived iTSC CM 14. Mass spectrometry-based proteomics together with a reciprocal immunoprecipitation predicted the interaction of Mtdh with MSN and ENO1, and RNA interference with Mtdh siRNA supported Mtdh-mediated anti-tumor actions of MSN and ENO1. Notably, the overexpression of Mtdh in MDA-MB-231 breast cancer cells converted cancer cells into iTSCs, and their CM reduced the MTT-based viability of cohort MDA-MB-231 cells. Furthermore, the application of extracellular PABPC1 reduced the level of Mtdh in MDA-MB-231 cells. Mtdh is a single-pass membrane protein that is located in the endoplasmic reticulum membrane, nucleus membrane, and cell junction 31. It can activate NFκB signaling 32 and promote chemoresistance and metastasis of breast cancer 33. Further analysis should test whether extracellular ENO1 and MSN may directly interact with Mtdh at the intercellular junctions and block Mtdh's tumorigenic action, or they may indirectly block tumorigenic signaling such as an NFκB regulatory axis via Mtdh.

While the administration of engineered T cells is clinically conducted, the use of their CM for any cancer treatment is not a current practice. The unique feature of CM is its integrity with thousands of protein species, but the potential advantage of this uniqueness may present difficulty in FDA approval and clinical practice. As an alternative option, the combination of multiple tumor-suppressing proteins such as PABPC1, ENO1, MSN, etc. can be considered a protein-based cocktail therapy. The cocktail proteins can be selected based on their combined efficacy as anti-tumor agents and the combination may differ for individual patients. We may employ MSCs as iTSC-generating host cells because of their application in regenerative medicine 34. Alternatively, we presented herein the possibility of using the peripheral blood from a patient with breast cancer for autologous CM generation. The additive effect of CM's anti-tumor action on a standard-of-care chemotherapeutic agent such as Taxol may facilitate clinical trials without harming the existing drug-based therapy.

This study has a few limitations. We presented herein the distinct anti-tumor action of AMPK-inactivated PBMC/lymphocyte-derived iTSC CM, CM's compatibility with Taxol in a mouse model of a mammary tumor, and the involvement of the Mtdh-mediated regulatory mechanism. First, CM generally contains varying molecules including nucleic acids, lipids, metabolites, etc. This study evaluated the role of extracellular proteins since nuclease treatment, exosome ultracentrifugation, and 3-kD cutoff filtering did not significantly alter the anti-tumor action of iTSC CM 6, 7. Second, while Dorsomorphin was employed to inactivate AMPK signaling, the agent is known as an inhibitor of bone morphogenetic protein signaling 35, 36. Third, the hormonal status of breast cancer may affect the significance of the ENO1/MSN-Mtdh regulatory axis in response to lymphocyte-derived iTSC CM. While the *ex vivo* assays in this study evaluated the positive responses to the engineered CM in the 5 subtypes of breast cancer, further examinations are necessary to establish statistically significant outcomes.

This study revealed the unconventional use of tumorigenic factors such as PABPC1, ENO1, and MSN as extracellular tumor-suppressing proteins, which were enriched in AMPK-inhibited PBMC/lymphocyte-derived CM. The anti-tumor action of ENO1 and MSN was mediated at least in part by Mtdh, a prognostic marker that substantially reduces the survival rate of cancer patients. Paradoxically, the overexpression of Mtdh in breast cancer cells converted cancer cells into iTSCs, which is the paramount facet of tumor-suppressing proteins in iTSCs. Since the described CM can downregulate PDL1, it might be possible to employ lymphocyte-derived iTSCs or their CM to promote CAR-T immunotherapy 37. In summary, we demonstrated that lymphocytes can be converted into iTSCs and their CM becomes an anti-tumor agent to prevent the progression of breast cancer, in a combinatorial application with Taxol. CM was also effective in blocking the development of osteoclasts and tumor-driven osteolysis. Further studies are warranted to evaluate the effectiveness of iTSC-derived protein-based options for the treatment of breast cancer and bone metastasis.

## Materials and Methods

**Cell culture and agents**. EO771 mouse mammary tumor cells (CH3 BioSystems, Amherst, NY, USA), 4T1.2 mouse mammary tumor cells (obtained from Dr. R. Anderson at Peter MacCallum Cancer Institute, Melbourne, Australia), J774A.1 mouse monocytes (obtained from Dr. Chien-Chi Lin at Indiana University Purdue University Indianapolis, IN, USA), MG63 and U2OS human osteosarcoma cell lines (#86051601-1VL and #92022711-1VL, Sigma, St. Louis, MO, USA), and PANC1 human pancreatic cells were cultured in DMEM. MDA-MB-231 breast cancer cells (ATCC, Manassas, VA, USA), and RAW264.7 pre-osteoclast cells (ATCC) were grown in αMEM. MDA-MB-436 and MCF-7 breast cancer cells (ATCC), and Jurkat T lymphocytes were cultured in RPMI1640. The culture media were supplemented with 10% fetal bovine serum and antibiotics (100 units/mL penicillin, and 100 µg/mL streptomycin; Life Technologies, Grand Island, NY, USA), and cells were maintained at 37 °C and 5% CO_2_.

Recombinant proteins for ENO1 (700 ng/mL, MBS2009113, MyBioSource, San Diego, CA, USA) and MSN (700 ng/mL, MBS2031729, MyBioSource) were given to MDA-MB-231 cells, and cells were incubated for 24 h. An AMPK inhibitor (Dorsomorphin, 50 µM, #3093, Tocris, Minneapolis, MN, USA) 38, an AMPK activator (AICAR, 50 µM, #2840, Tocris) 39, an activator of PKA signaling (CW008, 20 µM, #5495, Tocris) 40, and an activator of PI3K/Akt (YS49, 10 µM, #HY-15477, MedChem Express, NJ, USA) were applied to the cells for 24 h. Taxol (0.5 nM ~ 5 µM, #1097, Tocris) 41 was applied to breast cancer cells for 48 h.

**Preparation of CM.** For *in vitro* experiments, CM was subjected to low-speed centrifugation at 2,000 rpm for 10 min. The serum-free supernatants were centrifuged at 4,000 rpm for 10 min and subjected to filtration with a 0.22-μm polyethersulfone membrane (Sigma, St. Louis, MO, USA). To evaluate the effect of nucleic acids on the anti-tumor action of CM, we treated CM with nucleases for digesting DNA and RNA (#PI88700, Thermo Fisher Scientific, Waltham, MA, USA). For *in vitro* assays, the protein concentration of CM was adjusted to 100-250 μg/mL for PBMCs and ~100 μg/mL for monocytes. For examining the efficacy of CM *in vivo*, CM was condensed by a filter (3 kD cutoff), and the condensed CM (1 mg/mL, re-suspended in 50 µL of PBS) was intravenously injected from the tail vein.

**MTT and EdU assay**. MTT-based metabolic activity was evaluated using ~ 2,000 cells seeded in 96-well plates (Corning, Glendale, AZ, USA). CM was given on day 2, and cells were dyed with 0.5 mg/mL thiazolyl blue tetrazolium bromide (Sigma) on day 4 for 4 h. Optical density for assessing metabolic activities was determined at 562 nm using a multi-well spectrophotometer. Using the procedure previously reported 42, approximately 1,000 cells were seeded for the EdU assay in 96-well plates on day 1. CM was added on day 2 and cellular proliferation was examined with a fluorescence-based cell proliferation kit (Click-iT™ EdU Alexa Fluor™ 488 Imaging Kit; Thermo Fisher Scientific) on day 4. After fluorescent labeling, the number of fluorescently labeled cells was counted and the ratio to the total number of cells was determined.

**Transwell invasion assay**. In a transwell invasion assay, approximately 5×10^4^ cells in 300 µL serum-free DMEM were placed in the upper transwell chamber (Thermo Fisher Scientific) with Matrigel (100 µg/mL), and 800 µL of CM was added to the lower chamber. After 48 h, the cells that had invaded the lower side of the membrane were stained with Crystal Violet. At least five randomly chosen images were taken, and the average number of stained cells was determined.

**2D motility scratch assay**. A 2D dimensional motility scratch assay was conducted to evaluate 2-dimensional migratory behaviors. Approximately 4×10^5^ cells were seeded in 12-well plates. After cell attachment, a plastic pipette tip was used to scratch a gap in the cell layer. The floating cells were removed, and CM was added. Images of the cell-free scratch zone were taken at 0 h, and the areas newly occupied with cells were determined 24 h after the scratching. The areas were quantified with Image J (National Institutes of Health, Bethesda, MD, USA).

**Human peripheral blood mononuclear cells (PBMCs)**. Human peripheral blood samples were employed according to the guidelines of the Declaration of Helsinki, and their use was approved by the Ethics Committee of Osaka University (protocol #21344). We collected 8 mL of peripheral blood from 8 healthy volunteers (mean of 28 years, ranging from 24 to 33 years). Blood samples were diluted with an equal volume of 0.9% NaCl solution, and 6 mL of the diluted sample was mixed with 3 mL of a lymphoprep solution (#1114544, Abbott Diagnostics Technologies AS, Norway). Samples were centrifuged at 800×g for 30 min at room temperature, and PBMCs were collected with a Pasteur pipette. From PBMCs, monocytes were isolated using a forward and side scatter gating with FACS Aria Ⅱ flow cytometer (Becton, Dickinson and Company, Franklin Lakes, NJ, USA). PBMCs and monocytes were cultured in AlyS705 medium (Cell Science & Technology Institute, Inc., Sendai, Japan).

**Live-cell imaging.** A FRET-based biosensor was used to visualize AMPK activities in live cells. It was kindly provided by Dr. Jin Zhang (University of California San Diego, CA, USA) and Dr. Takanari Inoue (Johns Hopkins University, MD, USA), and its specificity has been extensively tested 43. For imaging experiments, cells were plated on a type I collagen-coated glass-bottom dish (MatTek, Ashland, MA, USA) and then transfected with the AMPK biosensor using Lipofectamine 3000 (Invitrogen, Waltham, MA, USA) according to the manufacturer's protocol. All the cells were maintained at 37˚C and 5% CO_2_ for 24-36 h before imaging experiments. A Nikon Ti-E inverted microscope with a 40× (0.75 numerical aperture) objective was used to visualize FRET images equipped with a filter wheel controller (Sutter Instruments, Novato, CA, USA), CFP excitation filter (438/24; Semrock, West Henrietta, NY, USA), CFP emission filter (483/32; Semrock), FRET emission filter (542/27; Semrock). Cells were illuminated with a 100 W Hg lamp through an ND64 (~1.5% transmittance) neutral density filter to minimize photobleaching. AMPK activity images were generated with NIS-Elements software (Nikon, Tokyo, Japan) by computing the emission ratio of FRET/CFP.

**Osteoclast differentiation assay**. The differentiation assay of RAW264.7 pre-osteoclasts was performed in a 12-well plate. During the 6-day incubation of pre-osteoclast cells in 40 ng/mL of RANKL, the CM was exchanged once on day 4. Adherent cells were fixed and stained with a tartrate-resistant acid phosphate (TRAP)-staining kit (Sigma), according to the manufacturer's instructions. TRAP-positive multinucleated cells (> 3 nuclei) were identified as mature osteoclasts.

**Plasmid transfection, RNA interference, and ELISA.** The overexpression of PABPC1, Mtdh, Lrp5, β-catenin, cMyc, and Akt was conducted by transfecting plasmids (#65807, #52331, #115907, #31787, #17758, #10841, respectively; Addgene, Cambridge, MA, USA), while blank plasmids (FLAG-HA-pcDNA3.1; Addgene) were used as a control. RNA interference was conducted using siRNA specific to Mtdh, CD44, and PABPC1 (#128411, #128413, s63659, s25666, respectively; Thermo Fisher Scientific) with a negative siRNA (Silencer Select #1, Thermo Fisher Scientific) as a nonspecific control using the procedure previously described 44. The expression levels of ENO1 and MSN in CM were detected by ELISA (MBS1604563, MBS2709503, MyBioSource).

**Western blot analysis**. Cultured cells were lysed in a radio-immunoprecipitation assay buffer and proteins were fractionated by 10-15% SDS gels and electro-transferred to polyvinylidene difluoride transfer membranes (Millipore, Billerica, MA, USA). The membrane was incubated overnight with primary antibodies and then with secondary antibodies conjugated with horseradish peroxidase (Cell Signaling, Danvers, MA, USA). Antibodies against p-Src, Src, Snail, cleaved-Caspase 3, Caspase 3, PDL1, ENO1, MSN, CD44, Mtdh, and PABPC1 (Cell Signaling), NFATc1, cathepsin K (Santa Cruz, Dallas, TX, USA) and β-actin as a control (Sigma) were employed. The level of proteins was determined using a SuperSignal west femto maximum sensitivity substrate (Thermo Fisher Scientific), and a luminescent image analyzer (LAS-3000, Fuji Film, Tokyo, Japan) was used to quantify signal intensities.

**Mass spectrometry-based proteomics analysis.** Mass spectrometry-based protein identification in CW008 and Dorsomorphin-treated Jurkat T lymphocyte-derived CM was conducted using the procedure previously described 6. The relative abundance was compared with control CM without any agent treatment. The top 50 protein candidates for each of the CW008 and Dorsomorphin treatments were listed (Table S1). Using MDA-MB-231 breast cancer protein samples that were immunoprecipitated with ENO1 and MSN, mass spectrometry-based global proteomics was also conducted using the procedure previously described 12. We used MS/MS counts to quantify relative protein levels and focused on proteins that had at least 2 MS/MS counts with one or more unique peptides. The top protein candidates (34 for ENO1, and 42 for MSN) were listed, together with their impact on the survival rate for patients with breast cancer and the rate with all cancer (Table S2). To evaluate the prediction by mass spectrometry-based proteomics analysis, we employed the recombinant proteins, including UBA1, SNRPE, UBE2L3, OXCT1, S100A9, PABPC1, LUM, COMP, APOB100 (MBS8121044, MBS7610177, MBS203071, MBS2033118, MBS9144157, MBS2123862, MBS145957, MBS144591, MBS2009355, respectively; MyBioSource). In the MTT assay, 5 µg/mL of each of these recombinant proteins was added and the viability of tumor cells was evaluated.

**Immunoprecipitation.** Immunoprecipitation was conducted with an immunoprecipitation starter pack kit (#45-000-369, Cytiva, Marlborough, MA, USA), using the procedure the manufacturer provided. Protein samples were pretreated with agarose beads conjugated with protein A and rabbit IgG, followed by overnight immunoprecipitation with the beads conjugated with anti-ENO1 antibodies, anti-MSN, or anti-Mtdh antibodies (Cell Signaling). The beads were collected by centrifugation, washed three times with PBS, and resuspended for Western blotting using antibodies against CD44, Mtdh, ENO1, and MSN (Cell Signaling).

***Ex vivo* tissue assay**. In the *ex vivo* tissue assay, the usage of human breast cancer tissues was approved by the Indiana University Institutional Review Board (#1911155674), and the tissues were received from Simon Cancer Center Tissue Procurement Core. A sample (~ 1 g) was manually fragmented with a scalpel into small pieces (0.5 ~ 0.8 mm in length), which were cultured in DMEM with 10% fetal bovine serum and antibiotics for a day. CM was then added for four additional days and a change in the size of at least four fragments was determined.

**Animal models**. The experimental procedures were approved by the Indiana University Animal Care and Use Committee (SC330R) and complied with the Guiding Principles in the Care and Use of Animals endorsed by the American Physiological Society. Mice were randomly housed in five per cage by a stratified randomization procedure based on body weight. Mouse chow and water were provided *ad libitum*. In the mouse model of a mammary tumor, NOD/SCID/γ (-/-) (NSG) female mice (~8 weeks) were provided by the *In Vivo* Therapeutics Core of the Indiana University Simon Comprehensive Cancer Center (Indianapolis, IN, USA). Mice were blindly divided into 3 groups (placebo, Jurkat CM, and Dorsomorphin-treated Jurkat CM groups; 8 mice per group). They received subcutaneous inoculation of MDA-MB-231 cells (3.0×10^5^ cells, 50 µL PBS) to the right side of the second pair in the mammary fat pad on day 1 45. From day 2, mice in the two treatment groups received a daily intravenous injection of Jurkat CM, and Dorsomorphin-treated Jurkat CM (50 µL of CM). The placebo mice received the same volume of PBS. For examining the additive anti-tumor effect of Taxol, mice were divided into 4 groups (placebo, Taxol, Dor CM, Taxol & Dor CM groups; 10 mice per group). They received a daily intravenous injection of Dorsomorphin-treated Jurkat CM (Dor CM group), a weekly intraperitoneal injection of Taxol (10 mg/kg) (Taxol group), and a daily intravenous injection of Dor CM with a weekly intraperitoneal injection of Taxol (Taxol & Dor CM group). The animals were sacrificed on day 18. Mammary tumors were isolated and their weights were measured.

In the mouse model of osteolysis (3 groups, 5 mice per group, ~8 weeks), NOD/SCID/γ (-/-) (NSG) mice received an injection of MDA-MB-231 breast cancer cells (2.5×10^5^ cells in 20 µL PBS), into the right tibia as an intra-tibial injection. The three treatment groups were placebo, Jurkat-derived CM, and Dorsomorphin-treated Jurkat-derived CM. CM was given daily as an intravenous injection to the tail vein. Mice were sacrificed after 18 days and the hindlimbs were harvested for microCT imaging and histology.

**X-ray imaging.** Whole-body X-ray imaging was performed using a Faxitron radiographic system (Faxitron X-ray Co., Tucson, AZ, USA) 46. Tibial integrity was scored in a blinded manner on a scale of 0-3: 0 = normal with no indication of a tumor, 1 = clear bone boundary with slight periosteal proliferation, 2 = bone damage and moderate periosteal proliferation, and 3 = severe bone erosion.

**μCT imaging and histology.** The tibiae were harvested for μCT imaging and histology. Micro-computed tomography was performed using Skyscan 1172 (Bruker-MicroCT, Kontich, Belgium) 47. Using manufacturer-provided software, scans were performed at pixel size 8.99 μm and the images were reconstructed (nRecon v1.6.9.18) and analyzed (CTan v1.13). Using µCT images, trabecular bone parameters such as bone volume ratio (BV/TV) and bone mineral density (BMD) were determined in a blinded fashion. In histology, H&E staining was conducted as described previously and images were analyzed in a blinded fashion 48. Of note, normal bone cells appeared in a regular shape with round and deeply stained nuclei, while tumor cells were in a distorted shape with irregularly stained nuclei.

**Statistical analysis.** For cell-based experiments, three or four independent experiments were conducted, and data were expressed as mean ± S.D. In animal experiments, we employed at least 5 mice per group to obtain statistically significant differences in tumor weight for testing mammary tumors and determining the four bone parameters. Statistical significance was evaluated using a one-way analysis of variance (ANOVA). Post hoc statistical comparisons with control groups were performed using Bonferroni correction with statistical significance at *p* < 0.05. The single and double asterisks in the figures indicate *p* < 0.05 and *p* < 0.01, respectively.

## Supplementary Material

Supplementary figures and table.

## Abbreviations

AMPK: 5'-AMP-activated protein kinase
ANOVA: analysis of variance
BMD: bone mineral density
BV/TV: bone volume ratio
Cat K: Cathepsin K
CM: conditioned medium
DMEM: Dulbecco's modified Eagle's medium
Dor: Dorsomorphin
EdU: 5-ethynyl-2'-deoxyuridine
ENO1: enolase 1
FRET: fluorescence resonance energy transfer
FSC: forward-scattered light
H&E: hematoxylin and eosin
iTS cells: induced tumor-suppressing cells
MSCs: mesenchymal stem cells
MSN: Moesin
Mtdh: Metadherin
OXCT1: oxoacid CoA-transferase 1
PABPC1: polyA-binding protein 1
PBMCs: peripheral blood mononuclear cells
PKA: Protein kinase A
S100A9: S100 calcium-binding protein A9
SNRPE: small nuclear ribonucleoprotein polypeptide E
SSC: side-scattered light
Tb.N: trabecular number
Tb.Sp: trabecular separation
Uba1: ubiquitin-like modifier activating enzyme 1
Vdac1: voltage-dependent anion-selective channel 1
