# Supplementary material for: Proteomes from AMPK-inhibited peripheral blood mononuclear cells suppress the progression of breast cancer and bone metastasis
Source: Theranostics. 2023 Feb 5;13(4):1247–63. doi: 10.7150/thno.80294 (PMC10008730; doi:10.7150/thno.80294)
Supplement: Supplementary file 1 — Supplementary figures and table. [file thnov13p1247s1.pdf]

Supplementary materials

Supplementary Table 1. Proteins enriched in lymphocyte-derived CM in response to the treatment with Dorsomorphin (AMPK inhibitor) and CW008 (PKA activator).

Gene	Description	Dor*	Gene	Description	CW008*
ACCS	Probable inactive 1-aminocyclopropane-1-carboxylate synthase-like protein 2	3.08	Uba1	Ubiquitin-like modifier-activating enzyme 1	4.60
CADM1	Cell adhesion molecule 1	2.79	SNRPE	Small nuclear ribonucleoprotein E	3.89
LUM	Lumican	2.73	UBR2L3	Ubiquitin-conjugating enzyme E2 L3	3.84
GSN	Gelsolin	2.65	HINT1	Adenosine 5'-monophosphoramidase HINT1	3.81
IGHEP2	Inter-alpha-trypsin inhibitor heavy chain H2	2.63	CLIC1	Chloride intracellular channel protein 1	3.68
XPO1	Exportin-1	2.62	SF1	Splicing factor 1	3.67
VNN1	Pantetheinase	2.56	OXCT1	Succinyl-CoA:3-ketoacid coenzyme A transferase 1, mitochondrial	3.46
LTF	Lactotransferrin	2.45	ALMS1	Alstrom syndrome protein 1	3.41
HYPK	Huntingtin-interacting protein K	2.28	RAD23B	UV excision repair protein RAD23 homolog B	3.31
SLC15A2	Solute carrier family 15 member 2	2.25	CDC42	Cell division control protein 42 homolog	3.05
GC	Vitamin D-binding protein	2.23	ANP32B	Acidic leucine-rich nuclear phosphoprotein 32 family member B	2.98
RBP4	Retinol-binding protein 4	2.22	Ezrin	Ezrin	2.95
OXCT1	Succinyl-CoA:3-ketoacid coenzyme A transferase 1, mitochondrial	2.20	CTSD	Cathepsin D	2.86
CAPRIN1	Caprin-1	2.16	HNRNPH1	Heterogeneous nuclear ribonucleoprotein H	2.79
COMP	Cartilage oligomeric matrix protein	2.14	Transgelin 2	Transgelin-2	2.78
ITIH1	Inter-alpha-trypsin inhibitor heavy chain H1	2.06	VNN2	Pantetheine hydrolase VNN2	2.78
PAIP1	Polyadenylate-binding protein 1	2.04	PDEF	Pigment epithelium-derived factor	2.76
VNN2	Pantetheine hydrolase VNN2	2.04	PSMB6	Proteasome subunit beta type-6	2.72
C3	Complement C3	2.03	S100A9	Protein S100-A9	2.72
UBA1	Ubiquitin-like modifier-activating enzyme 1	1.98	PNP	Purine nucleoside phosphorylase	2.68
TTR	Transthyretin	1.98	TUBB4B	Tubulin beta-4B chain	2.66
PBXIP1	Pre-B-cell leukemia transcription factor-interacting protein 1	1.94	PABPC1	Polyadenylate-binding protein 1	2.66
PSMB6	Proteasome subunit beta type-6	1.93	YWHAG	14-3-3 protein gamma	2.64
BAF	Barrier-to-autointegration factor	1.89	PRKCSH	Glucosidase 2 subunit beta	2.64
FN1	Fibronectin	1.87	DUT	Deoxyuridine 5'-triphosphate nucleotidohydrolase, mitochondrial	2.62
TPRA1	Transmembrane protein adipocyte-associated 1	1.87	ILF2	Interleukin enhancer-binding factor 2	2.61
AFP	Alpha-fetoprotein	1.87	CYCYBP	Calcyclin-binding protein	2.55
APOH	Beta-2-glycoprotein 1	1.87	CTBP1	C-terminal-binding protein 1	2.54
APOB	Apolipoprotein B-100	1.86	CSNK2B	Casein kinase II subunit beta	2.54
SERPINF2	Alpha-2-antiplasmin OS=Homo sapiens	1.86	ACTN4	Alpha-actinin-4	2.50
SERPINC1	Antithrombin-III	1.84	G6PD	Glucose-6-phosphate 1-dehydrogenase	2.50
SNRPE	Small nuclear ribonucleoprotein E	1.83	Thioredoxin	Thioredoxin	2.47
S100A9	Protein S100-A9	1.81	CD44	CD44 antigen	2.46
SERPINA7	Thyroxine-binding globulin	1.80	LUM	Lumican	2.43
CTBP1	C-terminal-binding protein 1	1.79	AARS1	Alanine--tRNA ligase, cytoplasmic	2.43
GLRX	Glutaredoxin-1	1.79	DDX17	Probable ATP-dependent RNA helicase DDX17	2.43
ESD	S-formylglutathione hydrolase	1.79	NASP	Nuclear autoantigenic sperm protein	2.41
Zpi	Protein Z-dependent protease inhibitor	1.78	RACK1	Receptor of activated protein C kinase 1	2.41
UBE2L3	Ubiquitin-conjugating enzyme E2 L3	1.76	HNRNPU	Heterogeneous nuclear ribonucleoprotein U	2.40
LSM5	U6 snRNA-associated Sm-like protein LSm5	1.75	SPTBN1	Spectrin beta chain, non-erythrocytic 1	2.39
CD44	CD44 antigen	1.74	HNRNPC	Heterogeneous nuclear ribonucleoprotein C-like 1	2.37
NAP1L1	Nucleosome assembly protein 1-like 1	1.74	eIF3	Eukaryotic translation initiation factor 3 subunit I	2.35
PSAP	Prosaposin	1.74	PI16	Peptidase inhibitor 16	2.33
SKP1	S-phase kinase-associated protein 1	1.73	DPYSL2	Dihydropyrimidinase-related protein 2	2.32
ITIH3	Inter-alpha-trypsin inhibitor heavy chain H3	1.71	DDX5	Probable ATP-dependent RNA helicase DDX5	2.27
PLG	Plasminogen	1.70	srsf3	Serine/arginine-rich splicing factor 3	2.27
Pairbp1	Plasminogen activator inhibitor 1 RNA-binding protein	1.69	MIF	Macrophage migration inhibitory factor	2.23
ALMS1	Alstrom syndrome protein 1	1.64	COMP	Cartilage oligomeric matrix protein	2.22
FBLL1	rRNA/tRNA 2'-O-methyltransferase fibrillarin-like protein 1	1.64	apoB100	Apolipoprotein B-100	2.15
RBM8A	RNA-binding protein 8A	1.64	PGAM1	Phosphoglycerate mutase 1	2.15

*The level in Dorsomorphin or CW008-treated Jurkat T-lymphocytes is the relative abundance compared to that of the control conditioned medium. Dor = Dorsomorphin.

Supplementary Table 2. Immunoprecipitated proteins with ENO1/MSN, and the p-values for the survival of breast cancer patients and all cancer patients.

Gene names	kDa	ENO1	p-value			Gene names	kDa	MSN	p-value	
			breast cancer	all cancer					breast cancer	all cancer
P4HB	57.1	89	0.460	0.001		P4HB	57.1	62	0.460	0.001
VIM	53.7	74	0.180	0.430		VIM	53.7	56	0.180	0.430
ATP5A1	59.8	51	0.780	0.000		ANXA2	38.7	34	0.034	0.590
ANXA2	38.7	44	0.290	0.000		GOT2	47.4	20	0.034	0.630
C1QBP	31.0	33	0.140	0.000		C1QBP	31.0	14	0.140	0.000
GOT2	47.4	31	0.034	0.590		PDIA6	48.1	14	0.250	0.000
PDIA6	48.1	23	0.250	0.000		EPB41l2	109.9	13	0.190	0.016
RTN4	126.6	22	0.064	0.013		ATP1A1	113.0	13	0.920	0.051
VDAC1	32.4	22	0.000	0.000		RTN4	126.6	12	0.064	0.013
EPB41l2	109.9	20	0.190	0.016		HMGB1	24.9	11	0.870	0.210
LRP1	504.7	20	0.680	0.000		GNB1	37.4	11	0.220	0.002
HMGB1	24.9	17	0.870	0.210		LRP1	504.7	10	0.680	0.000
ATP1A1	113.0	15	0.920	0.051		VDAC1	32.4	9	0.000	0.000
MYOF	233.3	10	0.340	0.000		MYOF	233.3	9	0.340	0.000
RAP1B	20.8	10	0.750	0.000		RAP1B	20.8	8	0.750	0.000
CKAP4	63.7	10	0.082	0.000		ITGB1	88.2	8	0.740	0.000
PHB2	33.3	10	0.210	0.130		GNB2l1	35.1	7	0.082	0.000
TFRC	85.7	9	0.700	0.000		CKAP4	63.7	7	0.610	0.000
GNAI2	40.5	9	0.470	0.005		IFITM3	15.0	7	0.610	0.000
EWSR1	68.5	9	0.440	0.380		TFRC	85.7	6	0.700	0.000
STOML2	38.4	8	0.950	0.210		STOML2	38.4	6	0.950	0.210
RAB5C	23.4	8	0.790	0.001		RAB5C	23.4	6	0.790	0.001
TMED10	24.9	8	0.770	0.004		GNAIL2	40.5	5	0.470	0.005
S100A10	11.2	8	0.800	0.000		EWSR1	68.5	5	0.440	0.380
ITGB1	88.2	7	0.740	0.000		TMED10	24.9	5	0.770	0.004
IFITM3	15.0	7	0.610	0.000		RPSA	32.8	5	0.180	0.000
GNB2	37.3	7	0.460	0.120		RALB	23.3	5	0.022	0.001
GNB1	37.4	6	0.220	0.002		RAC1	21.5	5	0.180	0.000
RALB	23.3	6	0.022	0.001		PHB2	33.3	4	0.210	0.130
CTTN	61.2	6	0.470	0.750		GNB2	37.3	4	0.460	0.120
MTDH	63.8	5	0.004	0.000		EXOC1	101.9	4	0.140	0.003
RAC1	21.5	5	0.180	0.000		VAPA	27.9	4	0.640	0.000
EXOC1	101.9	5	0.140	0.003		SLC3A2	58.3	4	0.750	0.220
PHB	29.8	5	0.480	0.000		SLC7A5	55.9	4	0.005	0.000
						EZR	69.4	4	0.038	0.630
						CTTN	61.2	3	0.470	0.750
						ANXA1	38.7	3	0.150	0.000
						RHOG	21.3	3	0.900	0.000
						GJA1	43.0	3	0.150	0.094
						MTDH	63.8	2	0.004	0.000
						S100A10	11.2	2	0.800	0.000
						PHB	63.8	2	0.480	0.000

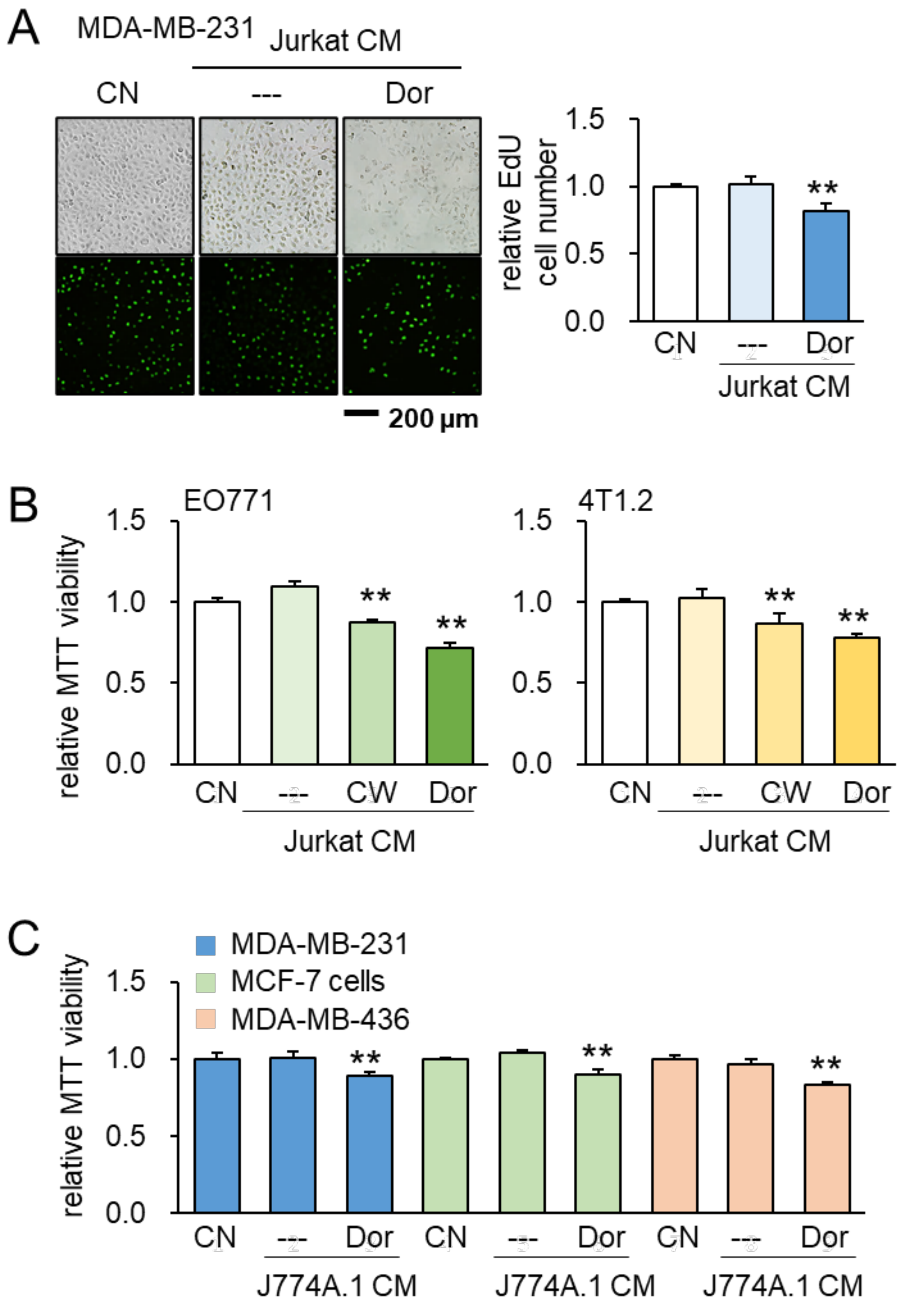

Suppl. Figure 1. **Tumor-suppressing CM from Jurkat lymphocytes and J774A.1 monocytes**. CN = control, Dor = Dorsomorphin (AMPK inhibitor), CW = CW008 (PKA activator), and CM = conditioned medium. The double asterisks indicate $p < 0.01$. (A) Reduction in EdU-based proliferation of MDA-MB-231 breast cancer cells by Jurkat cell-derived $CM^{Lym\text{-}Dor}$. (B) Reduction in MTT-based cell viability of EO771 and 4T1.2 mammary tumor cells by Jurkat cell-derived $CM^{Lym\text{-}Dor}$ and $CM^{Lym\text{-}CW}$, respectively. (C) Reduction in MTT-based cell viability of MDA-MB-231, MCF-7, and MDA-MB-436 breast cancer cells by J774A.1 monocyte-derived $CM^{mono\text{-}Dor}$.

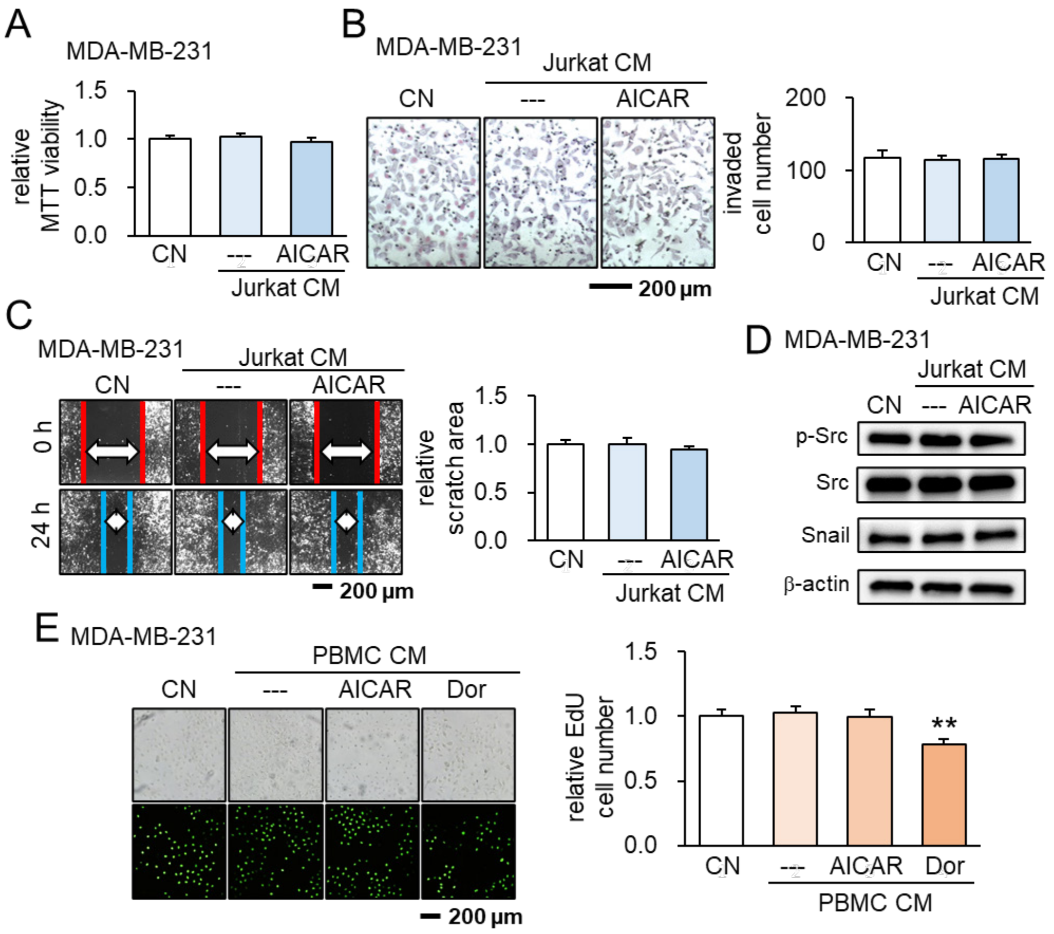

Suppl. Figure 2. **No detectable tumor-suppressive capability with CM$^{Lym\text{-}AICAR}$**. CN = control, CM = conditioned medium, PBMC = peripheral blood mononuclear cells, and Dor = Dorsomorphin (AMPK inhibitor). The double asterisks indicate $p < 0.01$. (A-C) No reduction in MTT-based viability, transwell invasion, and scratch-based migration of MDA-MB-231 breast cancer cells by CM$^{Lym\text{-}AICAR}$. Of note, AICAR is an activator of AMPK signaling. (D) No alteration in the expression level of p-Src and Snail in MDA-MB-231 cells by CM$^{Lym\text{-}AICAR}$. (E) No alteration in EdU-based proliferation of MDA-MB-231 breast cancer cells by mononuclear cell-derived CM$^{PBMC\text{-}AICAR}$, and reduction in EdU-based proliferation of MDA-MB-231 breast cancer cells by Jurkat cell-derived CM$^{PBMC\text{-}Dor}$.

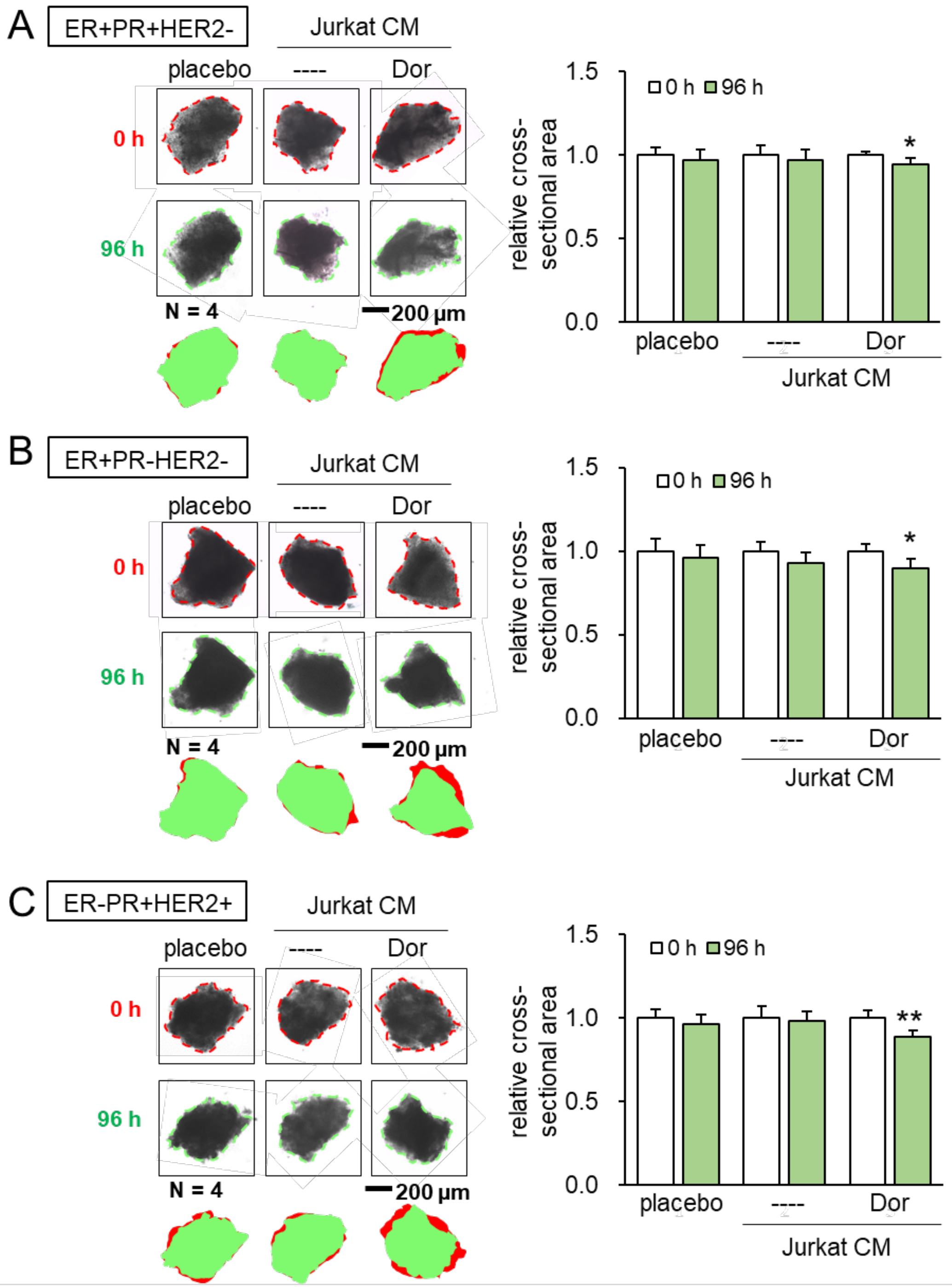

Suppl. Figure 3. **Shrinkage of human breast cancer tissue fragments by Jurkat lymphocyte-derived CM$^{Lym\text{-}Dor}$.** Dor = Dorsomorphin (AMPK inhibitor), and CM = conditioned medium. The single and double asterisks indicate $p < 0.05$ and 0.01, respectively. (A-C) Shrinkage of freshly isolated breast cancer tissues (ER+PR+HER2-, ER+PR-HER2-, and ER-PR+, HER2+), respectively.

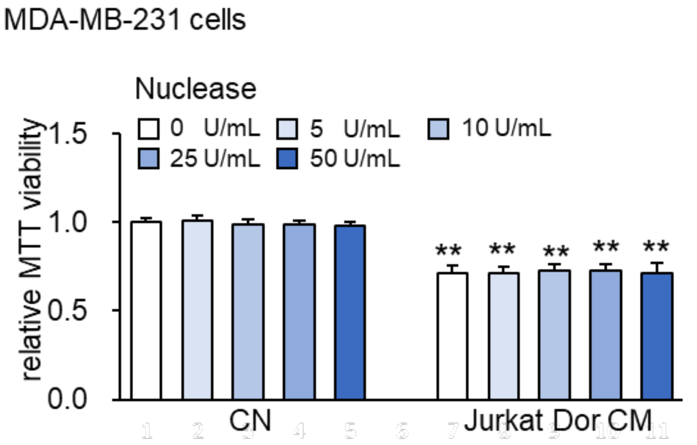

Suppl. Figure 4. No detectable effect of nuclease-treated CM[Lym-Dor] on MTT- based viability of MDA-MB-231 tumor cells. CN = control, Dor = Dorsomorphin (AMPK inhibitor), and CM = conditioned medium. The double asterisk indicates $p < 0.01$. The result suggests that anti-tumor ability is not induced by nucleic acids such as DNA and RNA.

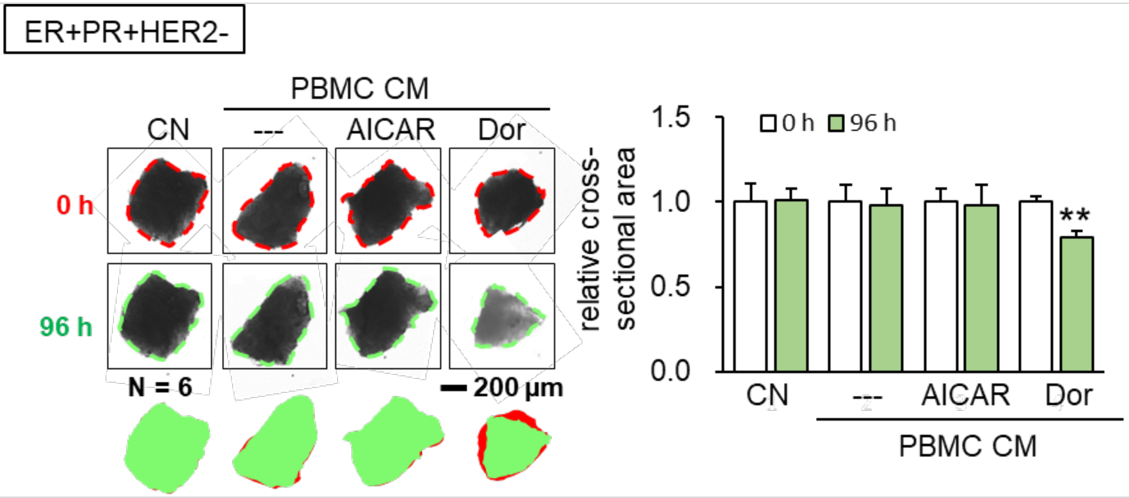

Suppl. Figure 5. Shrinkage of triple-negative human breast cancer tissue fragments by CM[PBMC-Dor], which was generated from the autologous peripheral blood. CN = control, Dor = Dorsomorphin (AMPK inhibitor), PBMC = peripheral blood mononuclear cells, and CM = conditioned medium. The double asterisks indicate $p < 0.01$. Shrinkage of freshly isolated breast cancer tissues (ER+PR+HER2-).

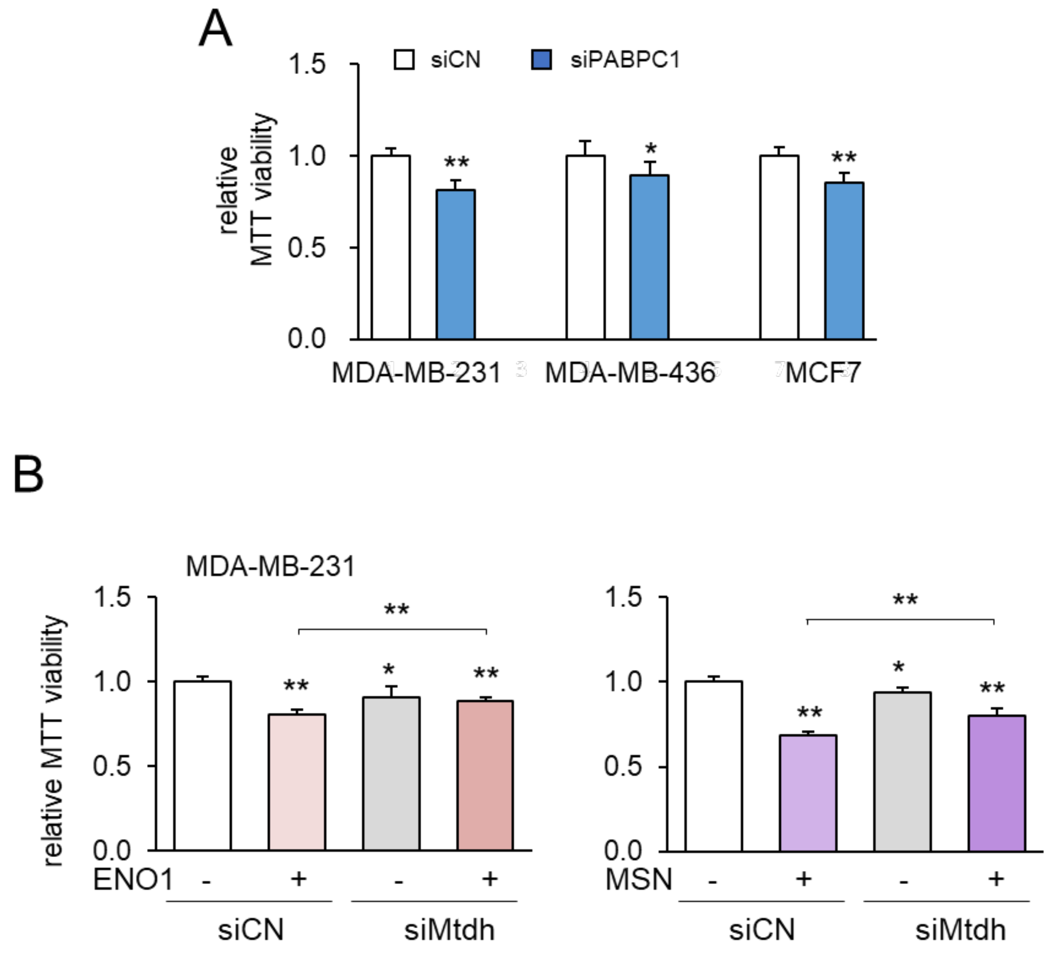

Suppl. Figure 6. **Effects of the second siRNA for PABPC1 and Mtdh**. siCN = control siRNA, siPABPC1 = PABPC1 siRNA, and siMtdh = Mtdh siRNA. The single and double asterisks indicate $p < 0.05$ and 0.01, respectively. (A) Reduction in MTT-based viability of MDA-MB-231, MDA-MB-436, and MCF-7 breast cancer cell lines by the silencing of PABPC1. (B) Reduction in the efficacy of ENO1 and MSN in Mtdh-silenced MDA-MB-231 cells.

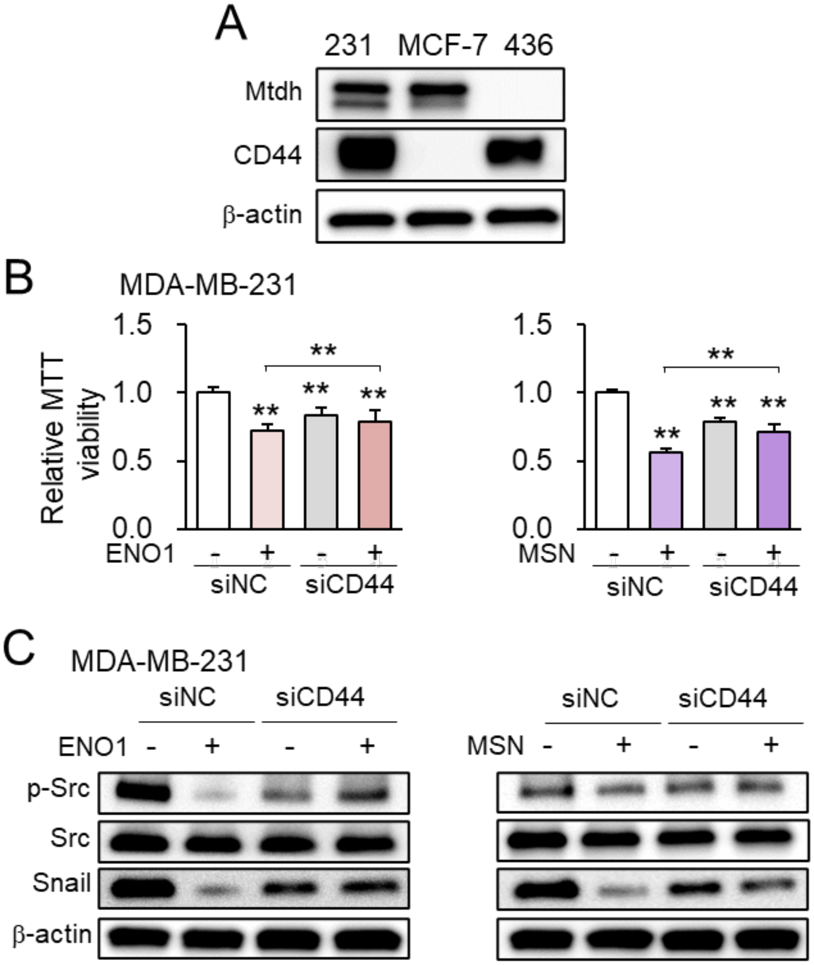

Suppl. Figure 7. Role of the Enolase 1 (ENO1)/Moesin (MSN)-CD44/Metadherin (Mtdh) regulatory axis. 231 = MDA-MB-231 breast cancer cells, 436 = MDA-MB-436 breast cancer cells, CN = control, CM = conditioned medium, NC = negative control, si = siRNA, and Dor = Dorsomorphin. The double asterisk indicates $p < 0.01$. (A) Expression of Mtdh and CD44 in three breast cancer cell lines. (B) Reduction in the efficacy of ENO1 and MSN in CD44-silenced MDA-MB-231 cells. (C) Reduction of p-Src and Snail by the application of ENO1 and MSN recombinant proteins, and its suppression by silencing CD44.

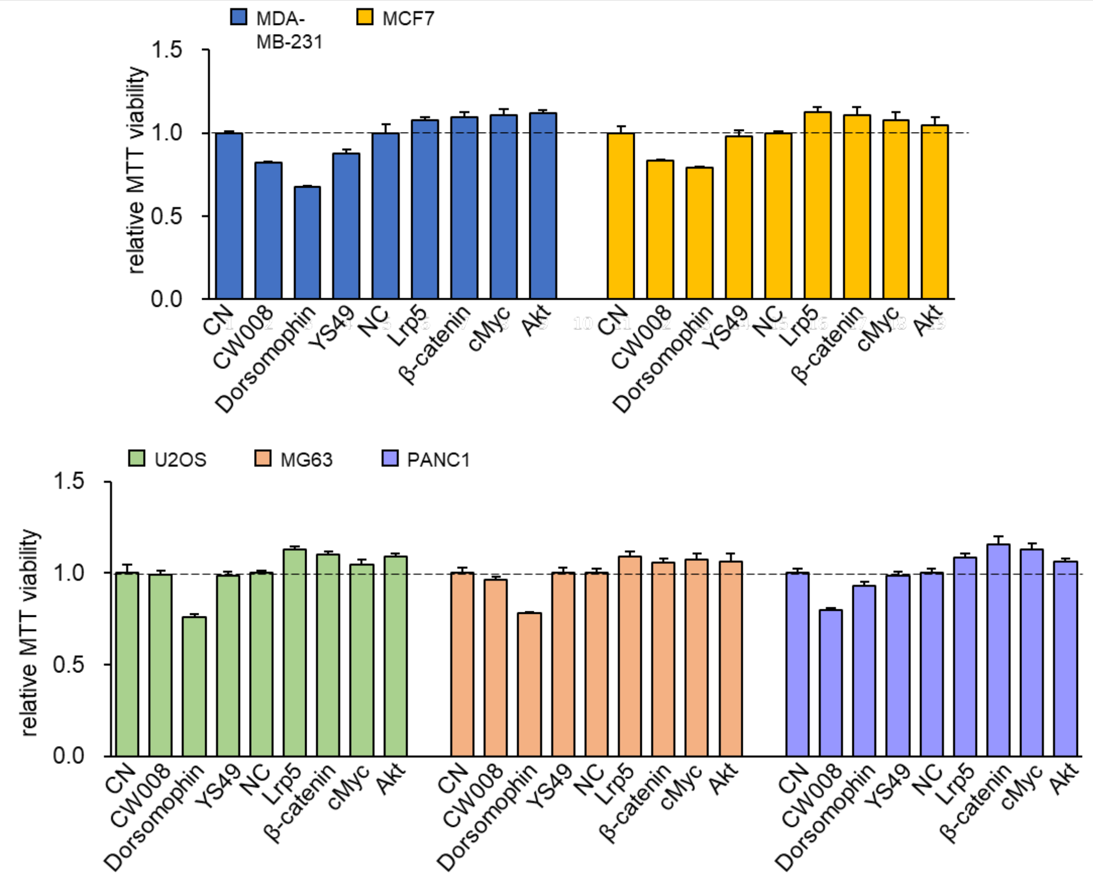

Suppl. Figure 8. MTT-based cell viability in response to CW008 (20 μM), Dorsomorphin (20 μM), YS49 (10 μM), and the overexpression of Lrp5, β-catenin, cMyc, and Akt in MDA-MB-231 breast cancer cells, MCF7 breast cancer cells, U2OS osteosarcoma cells, MG63 osteosarcoma cells, and PANC1 pancreatic cancer cells.
